# Longitudinal evaluation of a novel BChE PET tracer as an early *in vivo* biomarker in the brain of a mouse model for Alzheimer disease

**DOI:** 10.7150/thno.54589

**Published:** 2021-04-26

**Authors:** Luka Rejc, Vanessa Gómez-Vallejo, Ana Joya, Oscar Moreno, Ander Egimendia, Pilar Castellnou, Xabier Ríos-Anglada, Unai Cossío, Zuriñe Baz, Rossana Passannante, Ignacio Tobalina-Larrea, Pedro Ramos-Cabrer, Albert Giralt, Magdalena Sastre, Estibaliz Capetillo-Zarate, Urban Košak, Damijan Knez, Stanislav Gobec, Mariel Marder, Abraham Martin, Jordi Llop

**Affiliations:** 1 University of Ljubljana, Faculty of Chemistry and Chemical Technology, Večna pot 113, 1000 Ljubljana, Slovenia; 2 CIC biomaGUNE, Basque Research and Technology Alliance (BRTA), Paseo Miramon 182, 20014, San Sebastian, Spain.; 3 Laboratory of Neuroimaging and biomarkers of inflammation, Achucarro Basque Center for Neuroscience, Science Park UPV/EHU, 48940 Leioa, Spain; 4 Multiple Sclerosis Unit, Biodonostia Health Institute, Donostia-San Sebastián, Spain; 5 Department of Nuclear Medicine, University Hospital of Araba (HUA), 01009 Vitoria-Gasteiz, Spain.; 6 Department of Surgery Radiology and Physical Medicine, Faculty of Medicine, University of the Basque Country, UPV/EHU,01009 Vitoria-Gasteiz, Spain; 7 IKERBASQUE, Basque Foundation for Science, 48013 Bilbao, Spain; 8 Departament de Biomedicina, Facultat de Medicina i Ciències de la Salut, Institut de Neurociències. Barcelona, Spain.; 9 Institut d'Investigacions Biomèdiques August Pi i Sunyer (IDIBAPS), 08036 Barcelona, Spain.; 10 Centro de Investigación Biomédica en Red sobre Enfermedades Neurodegenerativas (CIBERNED), 28031 Madrid, Spain.; 11 Production and Validation Center of Advanced Therapies (Creatio), Faculty of Medicine and Health Science, University of Barcelona, 08036 Barcelona, Spain.; 12 Department of Brain Sciences, Imperial College London, Hammersmith Hospital, Du Cane Road, London W12 0NN.; 13 Department of Neuroscience, Faculty of Medicine and Nursery, University of the Basque Country UPV/EHU and Achucarro Basque Center for Neuroscience, Barrio Sarriena S/N, 48940 Leioa, Spain.; 14 University of Ljubljana, Faculty of Pharmacy, Aškerčeva cesta 7, 1000 Ljubljana, Slovenia.; 15 Universidad de Buenos Aires, Consejo Nacional de Investigaciones Científicas y Técnicas, and Instituto de Química y Fisicoquímica Biológicas, Facultad de Farmacia y Bioquímica, Universidad de Buenos Aires, Junín 956, C1113AAD Buenos Aires, Argentina; 16 Centro de Investigación Biomédica en Red - Enfermedades Respiratorias (CIBERES).

**Keywords:** Butyrylcholinesterase, PET, Positron Emission Tomography, Alzheimer Disease, Amyloid Beta.

## Abstract

**Purpose:** The increase in butyrylcholinesterase (BChE) activity in the brain of Alzheimer disease (AD) patients and animal models of AD position this enzyme as a potential biomarker of the disease. However, the information on the ability of BChE to serve as AD biomarker is contradicting, also due to scarce longitudinal studies of BChE activity abundance. Here, we report ^11^C-labeling, *in vivo* stability, biodistribution, and longitudinal study on BChE abundance in the brains of control and 5xFAD (AD model) animals, using a potent BChE selective inhibitor, [^11^C]**4**, and positron emission tomography (PET) in combination with computerised tomography (CT). We correlate the results with *in vivo* amyloid beta (Aβ) deposition, longitudinally assessed by [^18^F]florbetaben-PET imaging.

**Methods**: [^11^C]**4** was radiolabelled through ^11^C-methylation. Metabolism studies were performed on blood and brain samples of female wild type (WT) mice. Biodistribution studies were performed in female WT mice using dynamic PET-CT imaging. Specific binding was demonstrated by *ex vivo* and *in vivo* PET imaging blocking studies in female WT and 5xFAD mice at the age of 7 months. Longitudinal PET imaging of BChE was conducted in female 5xFAD mice at 4, 6, 8, 10 and 12 months of age and compared to age-matched control animals. Additionally, Aβ plaque distribution was assessed in the same mice using [^18^F]florbetaben at the ages of 2, 5, 7 and 11 months. The results were validated by *ex vivo* staining of BChE at 4, 8, and 12 months and Aβ at 12 months on brain samples.

**Results**: [^11^C]**4** was produced in sufficient radiochemical yield and molar activity for the use in PET imaging. Metabolism and biodistribution studies confirmed sufficient stability *in vivo*, the ability of [^11^C]**4** to cross the blood brain barrier (BBB) and rapid washout from the brain. Blocking studies confirmed specificity of the binding. Longitudinal PET studies showed increased levels of BChE in the cerebral cortex, hippocampus, striatum, thalamus, cerebellum and brain stem in aged AD mice compared to WT littermates. [^18^F]Florbetaben-PET imaging showed similar trend of Aβ plaques accumulation in the cerebral cortex and the hippocampus of AD animals as the one observed for BChE at ages 4 to 8 months. Contrarily to the results obtained by *ex vivo* staining, lower abundance of BChE was observed *in vivo* at 10 and 12 months than at 8 months of age.

**Conclusions:** The BChE inhibitor [^11^C]**4** crosses the BBB and is quickly washed out of the brain of WT mice. Comparison between AD and WT mice shows accumulation of the radiotracer in the AD-affected areas of the brain over time during the early disease progression. The results correspond well with Aβ accumulation, suggesting that BChE is a promising early biomarker for incipient AD.

## Introduction

Alzheimer Disease (AD) is the most common cause of dementia. It is one of the major causes of death globally and the second cause of death among the population over 70, affecting 13 million worldwide, the number expected to increase to more than 100 million by 2050 1. Currently, there is no effective treatment for AD available on the market, nor is there any reliable option for the early diagnosis of the disease. The diagnosis is therefore mainly based on clinical symptoms, which occur at later stages of the disease, when it is much too late for successful medical intervention 2. To overcome this growing problem and provide AD patients with better treatment options, there is an urgent need to identify new disease biomarkers to (i) enable longitudinal and non-invasive monitoring of disease progression, (ii) enable a targeted approach to design new treatment, diagnostic and theragnostic strategies by shedding light on the pathophysiology of AD and the mechanisms involved in disease progression, and (iii) provide with new tools for treatment response evaluation.

It has been described that neuronal loss contributes to cognitive and behavioural symptoms of AD 3, 4. Pathophysiologically, AD is characterized by the accumulation of amyloid-β (Aβ) aggregates in various conformations 5, the appearance of filamentous intraneuronal inclusions, dystrophic neurites and neurophilic threads whose main constituent is hyperphosphorylated Tau protein (p-Tau) 6, and synaptic dysfunction 7. AD patients also exhibit neuroinflammation 8, structural cerebrovascular alterations and early deficits in cerebral glucose uptake and cerebral blood flow responses 9. Some of these processes begin decades before the onset of clinical symptoms, constituting a unique opportunity for the early diagnosis of the disease and eventual evaluation of the response to treatment 10. In fact, the most recent trends propose establishing a definition of AD based on the assessment of different biomarkers, and not on clinical symptoms 2. The AT(N) scheme, recently published by the National Institute on Aging and Alzheimer's Association 2, proposes three groups of biomarkers, based on the nature of the pathological processes they measure: Aβ plaque (A), fibrillary tau (T) and neurodegeneration or neuronal damage (N) biomarkers. The AT(N) biomarkers can be investigated non-invasively *in vivo* by the means of positron emission tomography (PET) imaging, using radiotracers to detect Aβ and p-Tau 11, 12. This narrative shows how important the development of imaging tools based on different pathophysiological changes is; hence, out of the box search for new AD-specific biomarkers that would complement the existing diagnostic strategies is advantageous in order to obtain a reliable diagnostic method.

In addition to the changes above described, decreased enzymatic activity of acetylcholinesterase (AChE) and increased enzymatic activity of butyrylcholinesterase (BChE) was observed by biochemical analysis of tissue homogenates in brain regions where levels of Aβ and pTau deposits are usually increased 13. It seems that BChE takes over the role of AChE as the main enzyme for acetylcholine (ACh) deactivation 14, 15. This theory is confirmed by recent studies that show recovery of mice cognitive functions after scopolamine-induced short-term memory loss, by treatment with BChE inhibitors 16, 17. Furthermore, knock-out of BChE in the 5xFAD mouse model of AD showed a reduction of fibrillar Aβ plaque deposition 18, 19. These findings present a base for development of new imaging probes for the early or differential diagnosis of the disease, based on assessment of BChE levels in the brain.

First attempts to determine BChE abundance based on its enzymatic activity in the brains of AD patients by *in vivo* PET imaging resulted in moderate success 20. Contrary to prediction, BChE enzymatic activity decreased to 82 ± 14% in diseased individuals, suggesting that incremental BChE expression does not significantly contribute to ACh hydrolysis in AD, and that BChE was not a suitable target to distinguish AD from normal brain. The main reasons behind these negative results remain unclear, although unspecific binding, short ligand-enzyme latencies or migration of labelled metabolites after enzymatic hydrolysis of the radiotracer may play a role. Additionally, BChE was observed to interact with Aβ, which could affect its enzymatic function 21. This could also explain low success of radiolabelled BChE substrates to visualize increase in levels of BChE in the reported study 20. Recently, *N*-methylpiperidin-4-yl 4-[^123^I]iodobenzoate, a radiotracer labelled with the gamma emitter iodine-123 and suitable to conduct single photon emission computed tomography (SPECT) imaging studies, was validated by autoradiography 22 and *in vivo* imaging 23. The tracer proved efficient in quantifying cerebral BChE enzymatic activity and distinguished AD from healthy, age-matched mouse brains, showing 1.5- and 2.0-fold differences (AD vs. wild type, WT) at 60 min post injection for the hippocampus and cerebral cortex, respectively, at the age of 11.1 ± 1.1 months. However, clinical SPECT has certain limitations with respect to PET, including lower sensitivity and spatiotemporal resolution 24. Noteworthy, no information on how BChE levels change with age was obtained from this study, disabling the evaluation of BChE as AD progression biomarker. Although other attempts to develop PET-suitable radiotracers have been made, to the best of our knowledge there has been no reports on successful high resolution assessment of BChE biodistribution *in vivo* 25.

In this work, we shed a light on new possibilities of using BChE as AD biomarker and discus pitfalls of detection and evaluation of this enzyme *in vivo* and *ex vivo* in a transgenic mouse model. With this purpose, we have radiolabelled a recently reported potent BChE inhibitor 16 with the positron emitter carbon-11 (^11^C), and validated its use as *in vivo* PET tracer in two stages. First, biodistribution, metabolism and blood-brain barrier (BBB)-crossing capacity was assessed in WT mice. In parallel, its capability to detect BChE levels in an AD mouse model was evaluated by *ex vivo* and *in vivo* PET-CT blocking studies, using mice with fully developed disease. Second, the potential of this radiotracer for the *in vivo* longitudinal investigation of BChE expression was evaluated using PET-CT in a mouse model of AD. The results were compared to Aβ deposition in the same mice, investigated using the validated radiotracer [^18^F]florbetaben 26, 27, to better evaluate the correlation of BChE with AD progression. Finally, the presence of both, BChE and Aβ, was confirmed by *ex vivo* tissue staining and/or immunohistochemistry (IHC).

## Materials and Methods

### General aspects and study design

Animal handling was conducted in accordance with the European Council Directive 2010/63/UE. All experimental procedures were approved by the Ethical Committee at CIC biomaGUNE and local authorities (authorization number: PRO-AE-SS-095).

For metabolism and biodistribution studies, female WT C57BL/6 mice (n = 6; age = 10 weeks at arrival; 12-14 weeks at use) were obtained from Charles River. For BChE *ex vivo* and* in vivo* blocking studies, female WT (C57BL/6) and 5xFAD (C57BL/6J-Tg(APPSwFlLon,PSEN1*M146L*L286V)6799Vas/Mmjax, n = 2) mice were bred and housed in the animal facility of the Faculty of Psychology at the University of Barcelona and transferred to CIC biomaGUNE at the age of 7 months. Mouse genotyping was performed by polymerase chain reaction (PCR) in ear biopsy samples as described elsewhere 28. For longitudinal imaging studies, female WT C57BL/6J × SJL/J F1 mice (n = 10; control group), and female transgenic hemizygous 5xFAD (B6SJL-Tg(APPSwFlLon,PSEN1*M146L*L286V)6799Vas/Mmjax) mice (n = 13; study group) were obtained from The Jackson Laboratory (Bar Harbor, ME, USA). Animals arrived to CIC biomaGUNE at the age of 8-10 weeks and were imaged at different time points (see below). Imaging was performed during light phase of the light-dark cycle. Six additional animals per group were sacrificed at the ages of 4 (n = 2 per group), 8 (n = 2 per group) and 12 (n = 2 per group) months of age and the brains harvested for *ex vivo* studies.

### Chemistry and radiochemistry

**Scheme 1:**
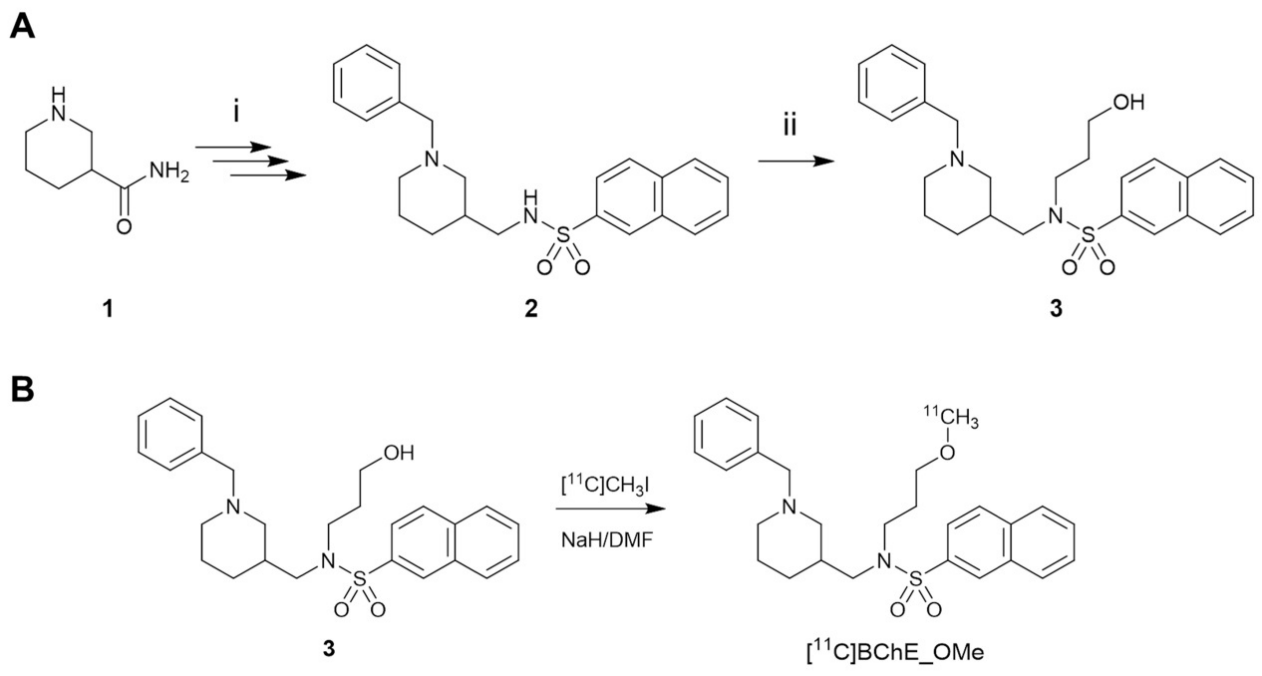
A) Reaction for the preparation of (±)*N*-[(1-benzylpiperidin-3-yl)methyl]-*N*-(2-hydroxyethyl)naphthalene-2-sulfonamide (3). Reagents and conditions: (i) see ref. 16; (ii) 2-bromoethane-1-ol, K_2_CO_3_, dry DMF, room temperature to 100 °C, 18 h, under argon; B) radiosynthesis of [^11^C]4 by ^11^C-methylation of the precursor 3.

The synthesis of [^18^F]florbetaben was performed using a TRACERlab FX_FN_ synthesis module (GE Healthcare) by ^18^F-fluorination of the *N*-BOC-protected precursor followed by hydrolysis, as previously described 29 (see ESI for details). The synthesis of the ^11^C-labelled BChE inhibitor (±)[^11^C]*N*-((1-Benzylpiperidin-3-yl)methyl)-*N*-(2-methoxyethyl)naphthalene-2-sulfonamide ([^11^C]**4**) (Scheme 1B) was achieved by ^11^C-methylation of (±)*N*-[(1-benzylpiperidin-3-yl)methyl]-*N*-(2-hydroxyethyl)naphthalene-2-sulfonamide **3** (prepared according to Scheme 1A; see ESI for experimental details). The hydrochloride salt of (±)*N*-((1-benzylpiperidin-3-yl)methyl)-*N*-(2-methoxyethyl)naphthalene-2-sulfonamide (**4**) was prepared as previously described 16.

### Metabolite analysis

For metabolite analysis, mice (n = 4) were injected intravenously (tail vein) with [^11^C]**4** under a general anaesthesia (5% isoflurane in pure O_2_ for induction; 1-2% isoflurane in pure O_2_ for maintenance; flow = 1 L/min). At pre-determined time points (5 and 15 min, n = 2 per time point) blood samples (ca. 200 µL) were obtained by cardiac puncture and animals were immediately perfused with physiologic saline solution. After complete perfusion, the brain was harvested. Blood samples were processed to separate the plasma, which was diluted with an equal volume of acetonitrile and centrifuged. The brain was homogenized in a glass homogenizer tube and acetonitrile in ultrapure water (1:1; 500 µL) was added. The supernatant from the blood plasma and the brain homogenates were analysed by HPLC (see ESI). The total sample process time after sacrifice was 10 min for blood plasma and 30 min for brain homogenates.

### PET-CT biodistribution studies

Whole body imaging studies (n = 2) to investigate biodistribution were conducted using PET in combination with computerized tomography (CT), using the β- and X-cube micro systems (Molecubes, Gent, Belgium). Anaesthesia was induced with 3.0-5.0% isoflurane in pure oxygen and maintained during imaging studies with 1.5-2.0% isoflurane in pure oxygen. PET scan started 30 ± 5 s after intravenous administration (tail vein) of 10% EtOH in saline solution containing [^11^C]**4** (5.1 ± 0.8 MBq; 0.029 ± 0.004 nmol; see Table S1, animals 28 and 29), to allow for animal assembly to PET bed and the scan initialization. Dynamic whole-body images were acquired list-mode in one bed position in a 511 keV ± 30% energetic window, with a total acquisition time of 90 min. The scan times were corrected for the delay in the PET experiment initialization. A CT scan was acquired immediately after finalisation of the PET imaging session. PET images were reconstructed with OSEM-3D iterative algorithm, using the following frames: 4 × 10 s, 4 × 30 s, 4 × 60 s, 4 × 120 s, 4 × 240 s, 3 × 900 s, 1 × 860 s. Images were analysed using π-MOD image analysis software (π-MOD Technologies Ltd, Zurich, Switzerland). Volumes of interest (VOIs) were manually drawn in major organs, namely brain, lungs, kidneys, liver, bladder and heart, using the CT images for anatomical reference, and decay-corrected time-activity curves were obtained.

### PET-CT blocking studies

BChE blocking PET-CT imaging studies were conducted to evaluate the specificity of compound **4** for the desired target, using β- and X-cube micro systems (Molecubes, Gent, Belgium). Anaesthesia was induced with 3.0-5.0 % isoflurane in pure oxygen and maintained during imaging studies with 1.5-2.0 % isoflurane in pure oxygen. Baseline PET scans started 30 ± 5 s after intravenous administration of 10% EtOH in saline solution containing [^11^C]**4** (see Table S1 for administered doses; animals 24-27). Blocking PET scans were acquired the following day on the same animals, by co-administering [^11^C]**4** (see Table S1) with hydrochloride salt of **4** (10 mg/kg; ca. 400 nmol per animal). In all cases, dynamic PET images were acquired in one bed position, with the brain centred in the middle of the field of view (FOV). The scan times were corrected for the delay in the PET experiment initialization. CT scans were acquired immediately after each PET acquisition. PET images were reconstructed with OSEM-3D iterative algorithm, using the following frames: 5 × 5 s, 5 × 10 s, 5 × 30 s, 5 × 60 s, 4 × 120, 4 × 240 s, 4 × 400 s. Images were analysed using π-MOD image analysis software. Volumes of interest were delineated in different brain regions (cerebral cortex, hippocampus, striatum, cerebellum, brain stem and thalamus) using the M. Mirrione-T2 MRI template. Uptake in the different regions was determined as standardized uptake values (SUV), and relative increase for each animal (non-blocked *vs*. blocked) was determined and expressed in percentage.

### PET-CT longitudinal brain studies

Longitudinal brain studies were performed using an eXplore Vista-CT small animal PET-CT system (GE Healthcare, WI, USA). The same WT and 5xFAD mice were used to study longitudinal accumulation of [^11^C]**4** and [^18^F]florbetaben in the brain. In all cases, anaesthesia was induced as described for biodistribution studies. Transgenic (AD) and age-matched control (WT) mice of the same genetic background were injected intravenously with [^11^C]**4** (8.5 ± 3.4 MBq; range: 1.4-16.1 MBq; 0.012-0.130 nmol; injected volume: 100-150 µL; 4, 6, 8, 10 and 12 months of age; see Table S1 for detailed injection list, animals 1-23) or [^18^F]florbetaben (15 ± 8 MBq; injected volume: 100-150 µL; 2, 5, 7 and 11 months of age). In case of [^11^C]**4**, PET scan started 30 ± 5 s after administration of the radiotracer, to allow for animal assembly to PET bed and the scan initialization. The dynamic PET images (frames: 5 × 5 s, 5 × 10 s, 5 × 30 s, 5 × 60 s, 4 × 120 s, 4 × 240 s, 4 × 400 s; total duration: 59.4 min) were acquired in one bed position, with the brain centred in the middle of the field of view (FOV). The scan times were corrected for the delay in the PET experiment initialization. In case of [^18^F]florbetaben, 30-min static PET images were acquired 30 min post intravenous injection in one bed position. CT scans (X-Ray energy: 40 kV; intensity: 140 µA) were acquired immediately after each PET acquisition. PET images were reconstructed using filtered back projection (FBP) applying random, scatter, and attenuation corrections and using a ramp filter with a cut off frequency of 1 Hz. Longitudinal deposition of BChE and Aβ was assessed in different brain regions (cerebral cortex, hippocampus, striatum, cerebellum, brain stem and thalamus for BChE; cerebral cortex, hippocampus, brain stem and cerebellum for Aβ) using the M. Mirrione-T2 MRI template available at π-MOD software. In case of [^11^C]**4**, time activity curves were obtained for different regions and expressed as standardized uptake values (SUV) to correct for injected dose and animal weight effects on the results. In case of [^18^F]florbetaben, the concentration of activity was determined in each region of interest (cortex, hippocampus and brain stem) as SUV value relative to the cerebellum (SUVr).

For the generation of [^11^C]**4** images, individual SUV images for each group (AD and WT) and time point were averaged. Voxel-by-voxel ratios of SUV values obtained at t = 6, 8, 10 and 12 months of age, with respect to t = 4 months, were displayed in different brain regions. For [^18^F]florbetaben, voxel-by-voxel SUVr values were determined in all brain regions, taking the average SUV value in the cerebellum as a reference. Images for data representation were created by voxel-by-voxel subtraction of SUVr values in the images obtained at t = 2 months from those obtained at t = 5, 7 and 11 months of age.

### *Ex vivo* studies

### Brain preparation

Animals were anesthetized with isoflurane, transcardially perfused with saline (20 mL, 0.9% NaCl, 0.01% sodium heparin) and the brain was removed, fast-frozen at -80 °C and cut in 25 µm- (*ex vivo* PET) or 10 µm- (staining and immunohistochemistry) thick sections in a cryostat and mounted on glass slides.

### Blocking studies

Brain slices (3 consecutive slices per repetition; Bregma values of *ca.* 1.2, -2.8 and -5.8) of 8 months old WT and AD animals (2 animals per strain and condition) were pre-incubated with PBS buffer (10 mM, pH = 7.4) for 15 min. Subsequently, samples were incubated (30 min, 25 °C) with 0.5 nM [^11^C]**4** both in the absence and presence of compound **4** (200 nM; autologous blocking) or ethopropazine (200 nM; BChE inhibitor with different chemical structure; heterologous blocking). After incubation, brain slices were rinsed twice with the same buffer and once with ultrapure water, dried at 50 °C and then measured using a β-cube micro system (Molecubes, Gent, Belgium). Images were reconstructed using OSEM-3D iterative algorithm, regions of interest (ROIs) were defined in the whole brain slices and the surface concentration of radioactivity for each slice was determined. The ratio in concentration of radioactivity (blocked/non-blocked) was determined for each group (WT and AD) and Bregma value, and expressed as percentage.

### Butyrylcholinesterase staining

Butyrylcholinesterase staining was performed using the Karnovsky-Roots method with modifications 30, 31. All reagents used in this protocol were purchased from Merck Life Science (Madrid, Spain). The substrate used for visualization of BChE activity was *S*-butyrylthiocholine iodide, and AChE activity was inhibited by BW284c51 (1,5-bis[4-allyl dimethylammonium phenyl]pentan-3-one dibromide) at a final concentration of 0.02 mM. In brief, tissue sections were rinsed in maleate buffer (0.1 M, pH 7.4) for 30 min and then incubated for 2 h in 0.1 M maleate buffer (pH 6.8) containing 0.5 mM sodium citrate, 0.47 mM cupric sulfate, 0.05 mM potassium ferricyanide, 0.8 mM *S*-butyrylthiocholine iodide and 0.02 mM BW284c51. All sections were then rinsed with gentle agitation for 30 min in dH_2_O and placed in 0.1% cobalt chloride in water for 10 min. After further rinsing in dH_2_O, 1 mL of filtered phosphate buffer containing 3,3'-diaminobenzidine tetrahydrochloride (DAB; 1.39 mM) was added to each slide. After 5 min in the DAB solution, 50 μL of 0.15% H_2_O_2_ in dH_2_O was added per ml of DAB solution, and the reaction was carried out for 20 min. Sections were then washed in 0.01 M acetate buffer (pH 3.3), dried overnight and mounted with Eukitt mounting medium for histology. Images were acquired with the Pannoramic MIDI II automated digital slide scanner (3DHistech Ltd., Hungary). For BChE inhibition, the same procedure was followed, but 0.032 mM ethopropazine hydrochloride was added to the maleate buffer to inhibit BChE activity.

### Amyloid immunohistochemistry

Amyloid plaques were visualized using immunohistochemistry for Aβ protein. Frozen sections were fixed with 4% paraformaldehyde for 15 min and rinsed three times for 5 min using 0.1 M PBS. Mouse on Mouse (M.O.M.^®^) peroxidase immunodetection kit was used following manufacture instructions (Vector Laboratories, CA, USA). In brief, endogenous enzyme activity was blocked with 3% H_2_O_2_ in 0.1 M PBS for 10 min followed by 0.1 M PBS washes. Mouse IgG was blocked with M.O.M blocking reagent for 1 h and 4G8 primary antibody (1:500) diluted in M.O.M diluent was incubated for 30 min. To obtain negative control images, the tissue slices were incubated with M.O.M diluent without primary antibody. VECSTAIN Elite ABC reagent was incubated for 5 min and sections were exposed for 6 min to peroxidase substrate solution (Vector Laboratories, CA, USA). After air dried, sections were mounted with PDX mountant for histology (Sigma, Mi, USA). Images were acquired with the Pannoramic MIDI II automated digital slide scanner (3DHistech Ltd., Hungary).

### Thioflavin staining

Thioflavin S staining was used to visualize fibrillar amyloid. Frozen sections were fixed with 4% paraformaldehyde for 15 min and rinsed with 0.1 M PBS 5 min 3 times. Sections were then incubated with 0.01% Thioflavin S in 70% ethanol, diluted 1:10 in 0.1 M PBS, for 10 min, washed with 0.1 M PBS and mounted with Fluoromont-G^®^ (SouthernBiothech, AL, USA). To obtain negative control images, the slices were incubated with 70% ethanol diluted 1:10 in 0.1 M PBS without Thioflavin S. Images were acquired with the Pannoramic MIDI II automated digital slide scanner (3DHistech Ltd., Hungary).

### Statistical analysis

PET results were analysed using two-way ANOVA. Differences between groups (5xFAD vs WT) at each time point and differences between time points within each group were determined using Sidak's multiple comparisons test. Differences were concluded significant for P values < 0. 05: P < 0.05, *; P < 0.01, **, P < 0.001, ***; and P < 0.0001, ****. Statistical tests were performed in GraphPad Prism 7.03 (GraphPad Software, CA, USA).

## Results

### Radiosynthesis and physico-chemical properties of [^11^C]4

By using the previously described captive solvent method 32 and in the presence of a strong base, sodium hydride, the radiosynthesis of [^11^C]**4** worked efficiently at room temperature in 5 min reaction time. Non-decay corrected radiochemical yields and molar activity values of 8 ± 4% and 161 ± 24 GBq/µmol, respectively, were obtained in overall synthesis time of 50 min. Radiochemical purity at injection time was >95% in all cases. Logarithm partition coefficient (logP) of [^11^C]**4** was 1.39 ± 0.03 (n = 3), suggesting that the polarity of the molecule is in the appropriate range to penetrate the blood brain barrier (BBB). This is in line with previous results that show BBB permeation ability for the non-labeled compound **4** using LC-MS/MS 16.

### Biodistribution and pharmacokinetics of [^11^C]4

**Figure 1:**
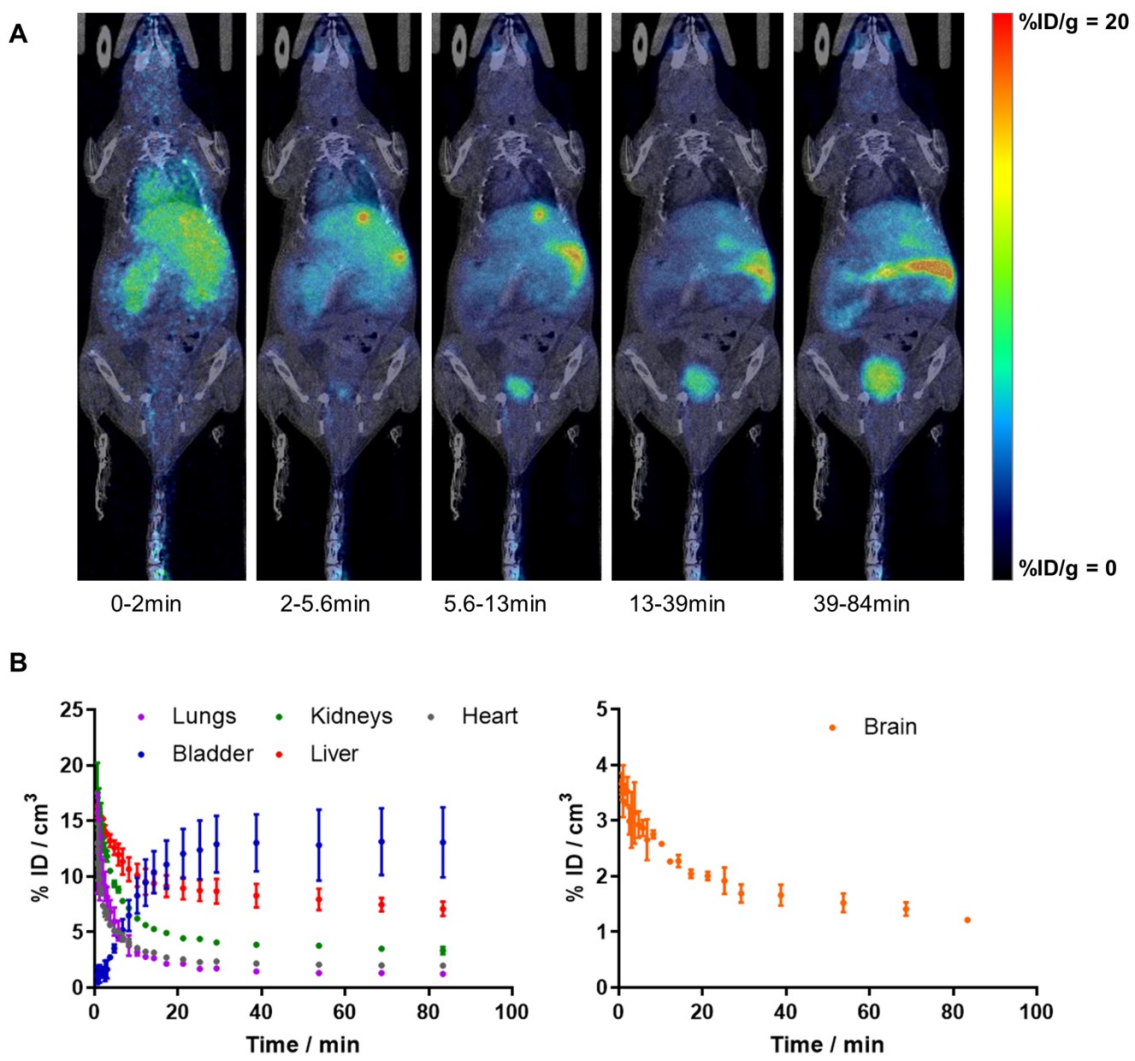
A) PET images (maximum intensity projections, coronal views) obtained at different time points after intravenous administration of [^11^C]4 in WT female mice. PET images have been co-registered with CT images (representative slices) of the same animal for proper localization of the radioactive signal; B) time activity curves in the lungs, bladder, kidneys, liver, heart and brain after intravenous administration of [^11^C]4 in WT female mice. The results are expressed and mean ± SD, n = 2.

Dynamic whole-body PET imaging studies (see Figure 1A for representative images; Figure 1B for time activity curves - TACs) performed in WT mice confirmed the BBB penetration capacity and subsequent clearance of [^11^C]**4**, with values of %ID/cm^3^ peaking at around t = 40 s (%ID/cm^3^ = 3.7 ± 0.1%) and slowly decreasing afterwards. Biodistribution data shows rapid clearance of radioactivity from the blood, as deduced from the fast decrease in activity concentration in the VOI drawn in the heart. TACs also show significant accumulation of radioactivity in the liver, and presence of radioactivity in the kidneys, which is paralleled by a progressive increase of radioactivity in the bladder, confirming elimination via hepatobiliary and urinary routes. Consistent with previously reported metabolite analysis 16, plasma analysis confirmed the presence of two radioactive polar metabolites with t_R_ of 2.0 (major metabolite) and 3.4 min (minor metabolite) under our chromatographic conditions (t_R_ for parent compound: 9.8 min). Altogether, metabolites accounted for 58% and 77% of the radioactivity in blood at t = 5 and 15 min after intravenous administration, respectively. Importantly, the presence of radioactive metabolites in the brain was lower, with values of 28% and 49% at t = 5 and 15 min after administration, respectively. Of these values, the metabolite with t_R_ = 2 min accounted for 90% of total metabolites.

### *In vivo* BChE blocking studies

**Figure 2:**
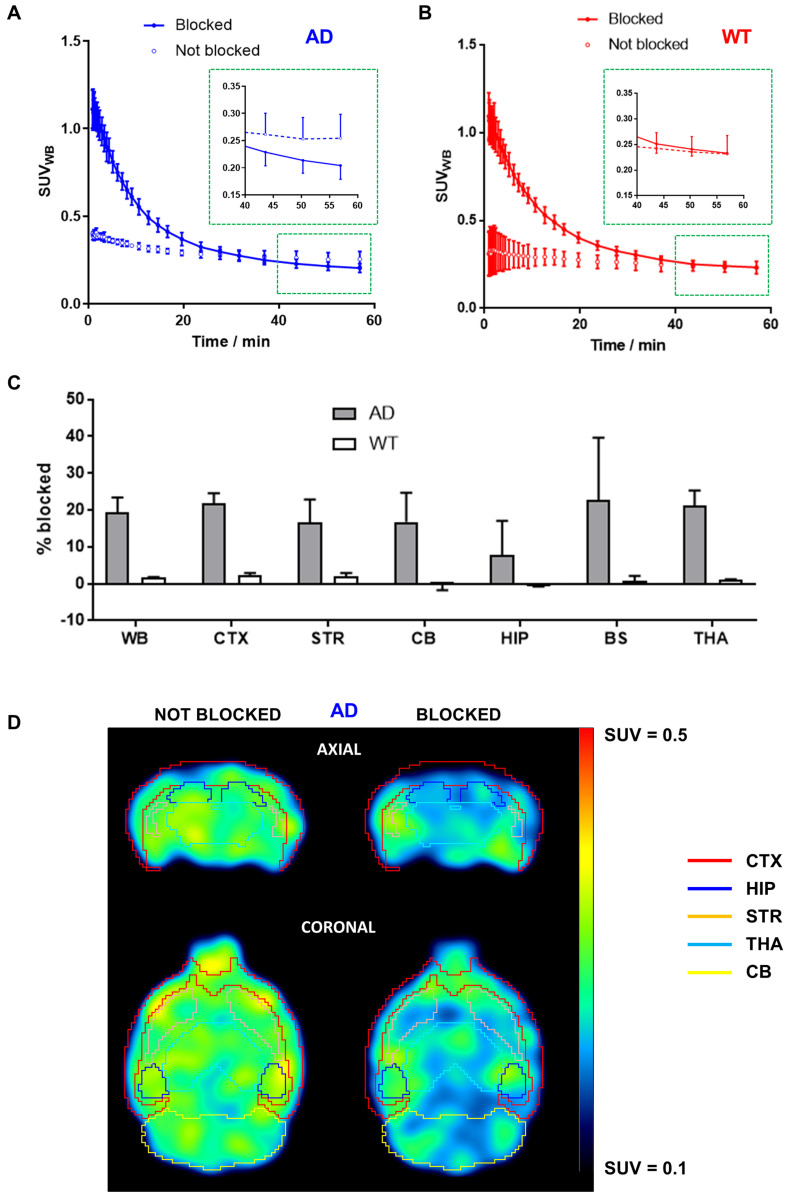
A, B) Time activity curves in the whole brain after intravenous administration of [^11^C]4 in AD (A) and WT (B) female mice. For blocking experiments (“blocked” in the figure) the radiotracer was co-injected with hydrochloride salt of compound 4 at a final dose of 10 mg/kg (injected amount of *ca.* 400 nmol per animal). For easier visualization of the uptake differences at the end of the blocking study, magnified graph of the last three time-points of PET imaging protocol (depicted by red square) is inset in A and B. Results are expressed as mean ± SD, n = 2; C) Percentage of blocking by co-administration of hydrochloride salt of 4 (dose = 10 mg/kg) in the whole brain (WB, considered as the sum of all brain regions segmented in the T2 Mirrione brain template provided by π-MOD software), cortex (CTX), striatum (STR), cerebellum (CB), hippocampus (HIP), brain stem (BS) and thalamus (THA), for both AD (grey bars) and WT (white bars) mice. Results are expressed as mean ± SD, n = 2; D) PET images (top: axial sections; bottom: coronal sections) obtained after administration of [^11^C]4 to AD mice under non-blocking (left) and blocking (right) conditions. Images correspond to the last time frame of the acquisition. Brain sub-regions are delineated for anatomical localization of the radioactive signal.

Significantly higher initial brain uptake of [^11^C]**4** was observed in animals injected with a solution containing hydrochloride salt of **4** in AD animals (Figure 2A). As indicated by biodistribution studies, the radiotracer rapidly cleared from the brain. Interestingly, the radiotracer levels at 50-60 min after injection were lower for BChE-blocked animals than in non-blocked animals (Figure 2A, inset), with a decrease in uptake of ca. 20% in brain sub-regions such as the cortex (21.7 ± 2.9%), striatum (16.7 ± 6.2%), cerebellum (16.8 ± 7.9%), brain stem (22.7 ± 17.0%), and thalamus (21.1 ± 4.2%) and slightly lower in the hippocampus (7.7 ± 9.2%; Figure 2C) at the last imaging time point. The lower uptake in animals treated with the mixture of hydrochloride salt of **4** and [^11^C]**4** was clearly visible on PET images (Figure 2D). Noteworthy, the concentration of radioactivity in the brain of WT animals under blocking conditions at the end of the imaging session was equivalent to the value obtained under non-blocking conditions (Figure 2B, inset), with differences in both experimental scenarios below 3%, irrespective of the brain sub-region (Figure 2C).

### *Ex vivo* BChE blocking studies

The concentration of radiotracer after incubation with [^11^C]**4** in the presence of either the hydrochloride salt of **4** or ethopropazine hydrochloride at a concentration of 200 nM was lower than the concentration of radioactivity under non-blocking conditions for AD animals, irrespective of the location within the brain. Blocked/non-blocked ratios of 93.0 ± 1.0% and 92.2 ± 1.6% (Bregma = 1.2; compound **4** and ethopropazine, respectively); 90.0 ± 2.6% and 87.2 ± 3.8% (Bregma = -2.8; compound **4** and ethopropazine, respectively); and 92.2 ± 0.8% and 88.9 ± 1.8% (Bregma = -5.8; compound **4** and ethopropazine, respectively) were obtained for AD animals (Figure S1). Contrarily, values close to 100% (meaning no-blocking effect) were obtained for WT animals: 99.7 ± 2.8 and 100.3 ± 3.5 (Bregma = 1.2; compound **4** and ethopropazine, respectively); 98.1 ± 2.2 and 99.1 ± 3.1 (Bregma = -2.8; compound **4** and ethopropazine, respectively); and 97.3 ± 0.7 and 98.4 ± 1.8 (Bregma = -5.8; compound **4** and ethopropazine, respectively). These values suggest a *ca.* 10% of specific binding in AD animals.

### Longitudinal imaging studies

**Figure 3:**
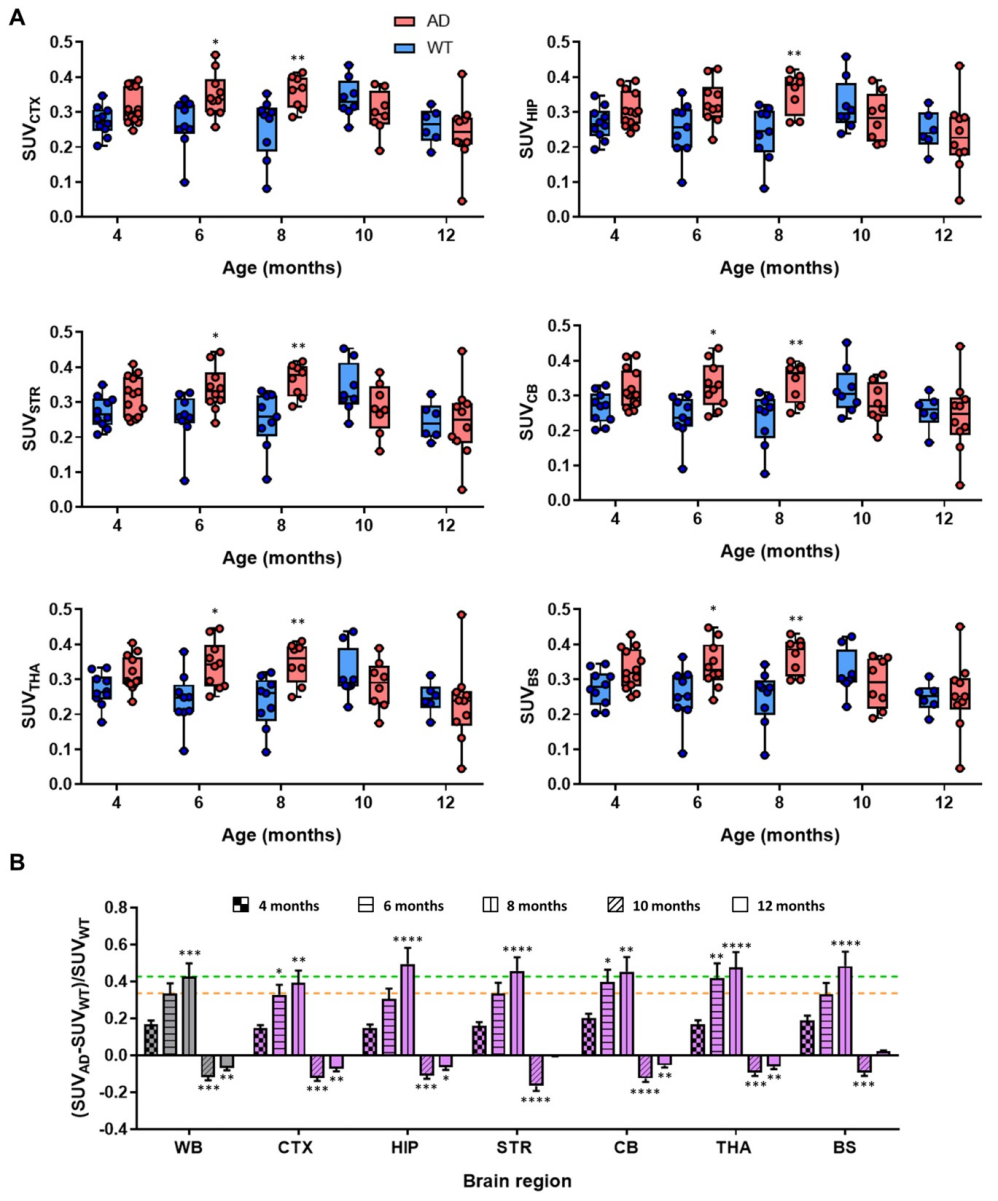
A) Box plot diagrams of SUV in different brain regions (whole brain: WB, considered as the sum of all brain regions segmented in the T2 Mirrione brain template provided by π-MOD software; cortex: CTX; hippocampus: HIP; striatum: STR; cerebellum: CB; thalamus: THA; and brain stem: BS) after intravenous administration of [^11^C]4 to AD and WT animals at the ages of 4, 6, 8, 10 and 12 months. For the calculation of SUV values, the last three frames of time activity curves for each region have been averaged; probability values (difference AD vs. WT) are depicted as * (P < 0.05), ** (P < 0.01); B) relative increased uptake of [^11^C]4 in different brain regions of AD animals with respect to WT age-matched animals, calculated as (SUV_AD_ - SUV_WT_)/SUV_WT_; yellow and green lines show increased uptake at the whole brain (WB) level at the ages of 6 and 8 months, respectively. Probability values (differences with respect to value at age = 4 months) are depicted as * (P < 0.05), ** (P < 0.01), *** (P < 0.001), and **** (P < 0.0001).

Dynamic PET-[^11^C]**4** imaging showed evident differences between AD and control mice. Initially, at age of 4 months, dynamic curves for AD and WT animals at the whole brain level were almost equivalent (Figure S2); however, later time points (6 and 8 months) reveal clear increase in the radiotracer uptake in AD compared to control mice (Figure S2). For easier comparison of the differences and to obtain regional information, time-activity curves were obtained for each brain region (CTX, HIP, STR, CB, THA, BS) and the last three time-frames (40-60 min after injection) of each scan were averaged and compared at different ages (Figure 3A). No significant difference in SUV and low intra-subject variability was observed between control and AD mice at the age of 4 months. Contrarily, the values in the cortex were significantly higher for AD animals than for aged-matched controls at 6 and 8 months of age (P values of 0.026 and 0.0098 for 6 and 8 months, respectively). Similar results were observed in other brain sub-regions e.g. striatum (P values of 0.049 and 0.0063 for 6 and 8 months, respectively), cerebellum (P values of 0.0193 and 0.0102 for 6 and 8 months, respectively), thalamus (P values of 0.0159 and 0.0102 for 6 and 8 months, respectively) and brain stem (P values of 0.049 and 0.0033 for 6 and 8 months, respectively) (Figure 3). For the hippocampus, significant differences with respect to control group were only observed at the age of 8 months (P = 0.0047). Interestingly, no significant differences were observed at the age of 10 and 12 months in any of the brain sub-regions. The observed trend is more evident when comparing the standardized differences in the radiotracer uptake between AD and WT mice [(SUV_AD_ - SUV_WT_)/SUV_WT_] (Figure 3B). Increase in relative uptake is in the range 0.30-0.43 at the age of 6 months and 0.40-0.48 at the age of 8 months.

**Figure 4:**
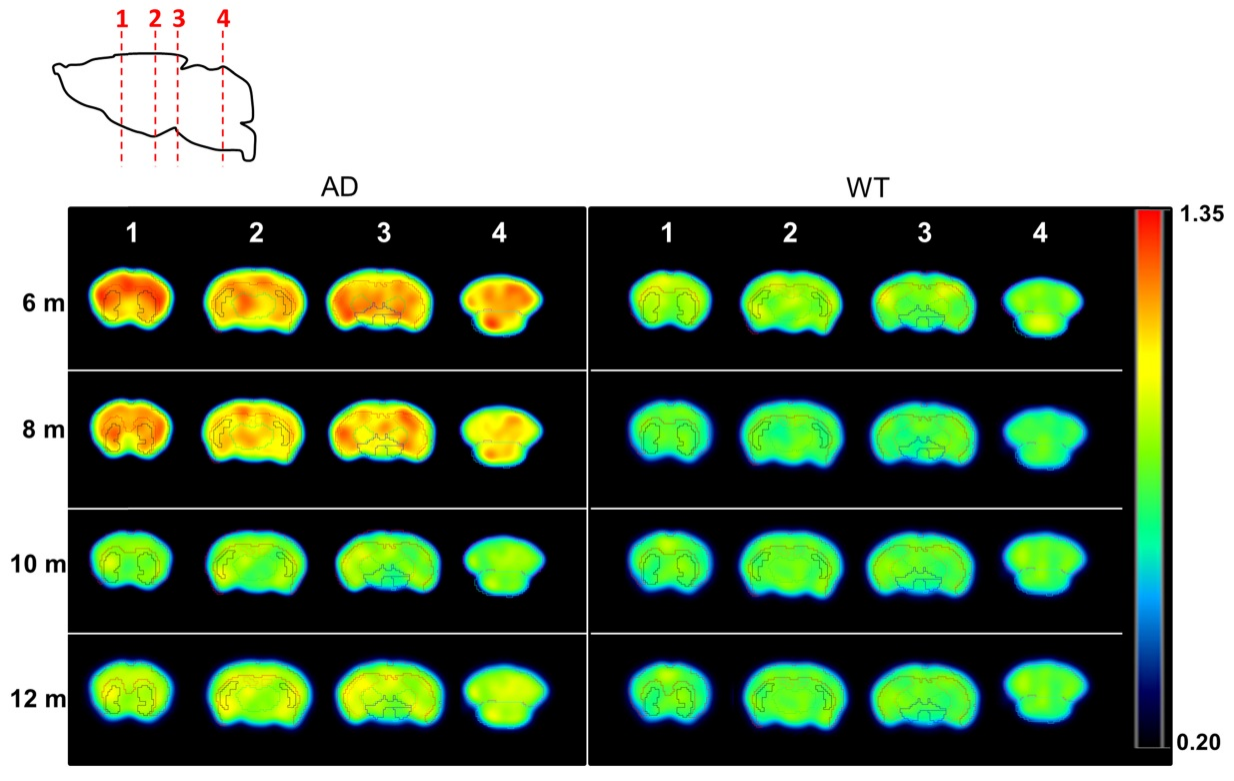
PET images (representative axial slices) obtained in AD and WT animals after intravenous administration of [^11^C]4 at different ages. Images have been generated by dividing, voxel-by-voxel, averaged images obtained at 6, 8, 10, and 12 months of age by averaged images obtained at 4 months of age. VOIs corresponding to the cortex, hippocampus, striatum, brain stem, thalamus and cerebellum are drawn for anatomical reference.

The differences between time points and animal groups are clearly visible in PET images when dividing average, voxel-by-voxel, radiotracer uptake at 6, 8, 10, and 12 months of age by 4 months of age in all the animals of each group (Figure 4). All brain regions considered in the study clearly show increased uptake at the ages of 6 and 8 months with respect to 4 months, while quasi-equivalent values in both WT and AD animals are obtained at 10 and 12 months of age.

**Figure 5:**
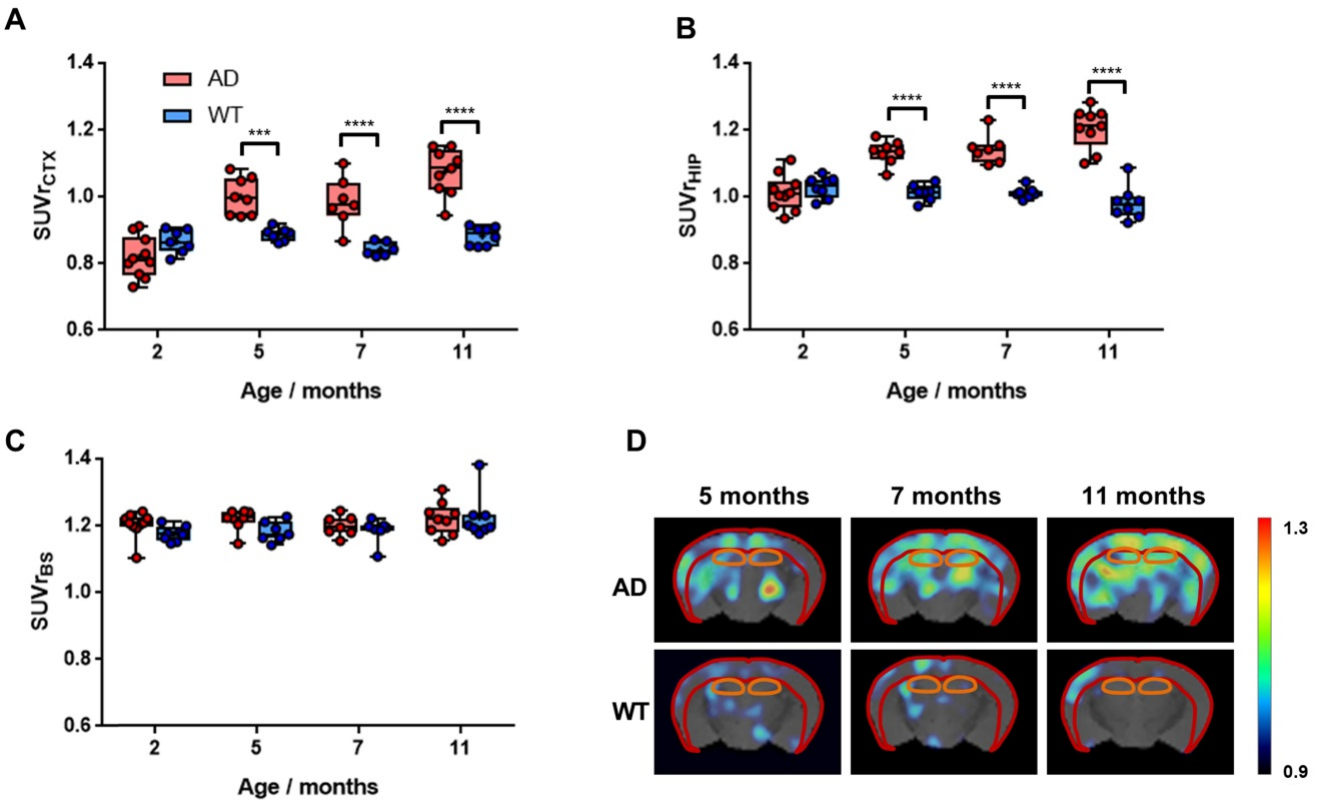
A-C) Box plot diagrams representing uptake of [^18^F]florbetaben in the cortex (CTX; A), the hippocampus (HIP; B) and the brain stem (BS; C) for WT and AD animals at different ages, expressed as Standard Uptake Values relative to the cerebellum (SUVr); dots represent individual values; probability values are depicted as * (P < 0.05), ** (P < 0.01), *** (P < 0.001), and **** (P < 0.0001); D) representative axial PET images corresponding to WT and AD animals at ages = 5, 7 and 11 months, representing, voxel-by-voxel, increased SUVr values with respect to the value at the age of 2 months for the same animal. PET images have been co-registered with a mouse brain atlas. Volumes of interest drawn in the cortex (red) and the hippocampus (orange) are displayed on each image.

Uptake values of [^18^F]florbetaben in different brain regions were calculated relative to the cerebellum, which was taken as the reference region, as no Aβ pathology has been reported in this brain region in 5xFAD animals 33. In the cortex, SUVr increased for AD animals from 2 to 5 months of age (P < 0.0001) to stabilize afterwards (Figure 5A). Progressive increase in SUVr values for AD animals over time was even more apparent in the hippocampus (Figure 5B). In WT animals, stable values over time were observed in both brain regions. When comparing values obtained for AD and age-matched WT animals, significant differences were observed at all ages in the cortex, with P values of 0.0004, <0.0001 and <0.0001 for 5, 7 and 11 months, respectively (see Table S2 for more detailed information). In the hippocampus, the differences were even more significant, with P values <0.0001 at all times. Such differences can also be observed on the images, obtained by calculating, voxel-by-voxel, the difference between the brain images at 5, 7, and 11 months of age and the image of the same animal at age of 2 months (Figure 5D). Contrarily, in the brain stem, a clear trend in uptake values vs. age could not be identified and differences with respect to the control group were not significant at any age (Figure 5C).

### Evaluation of BChE and Amyloid plaque staining

**Figure 6:**
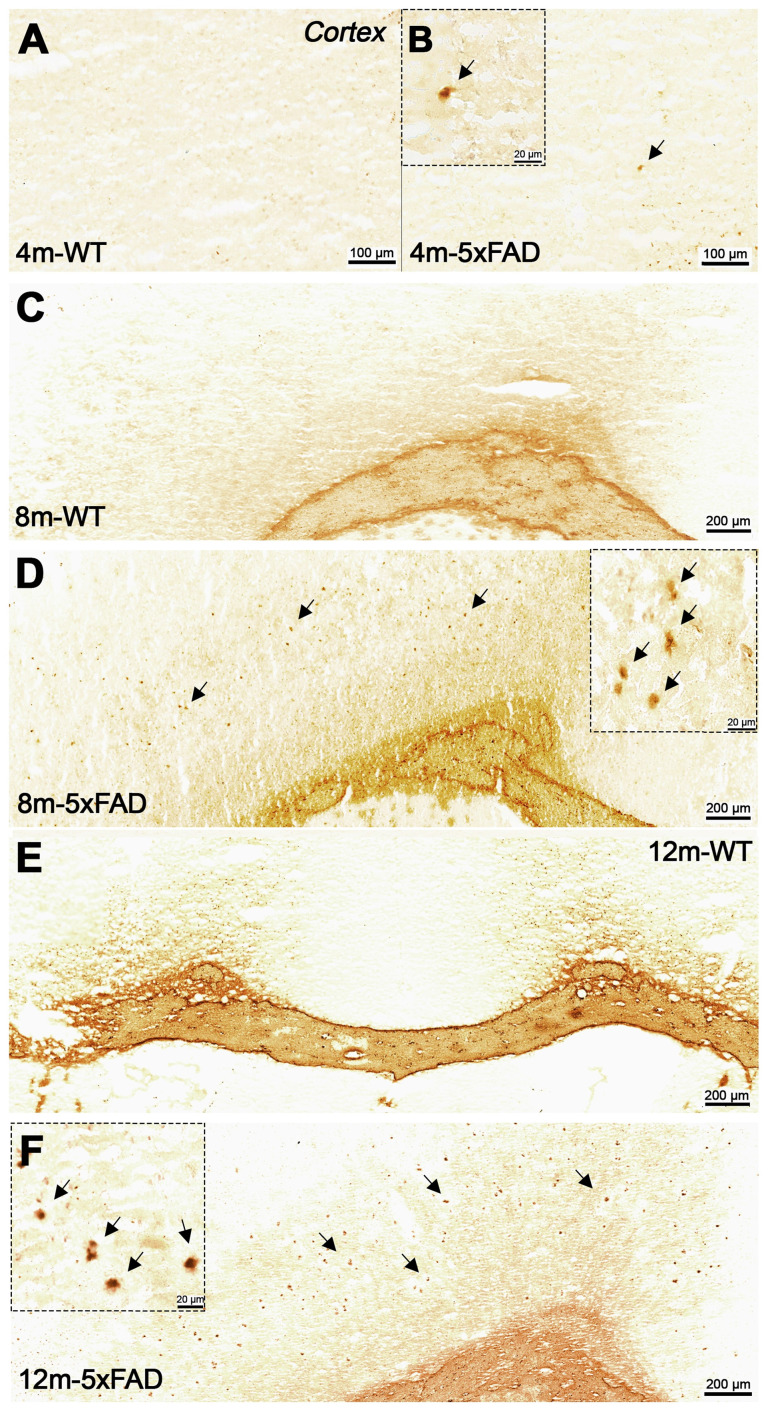
Staining for BChE enzymatic activity in 4, 8 and 12-month-old brains of WT (A, C and E) and 5xFAD mice (B, D and F) using the Karnovsky-Roots method. BChE staining showed increase of enzyme activity in the cerebral cortex of 5xFAD at different ages in comparison to WT (A to F) mice. Magnified images show the co-occurence of plaques with BChE enzyme activity in different regions of the cerebral cortex (B, D and F).

**Figure 7:**
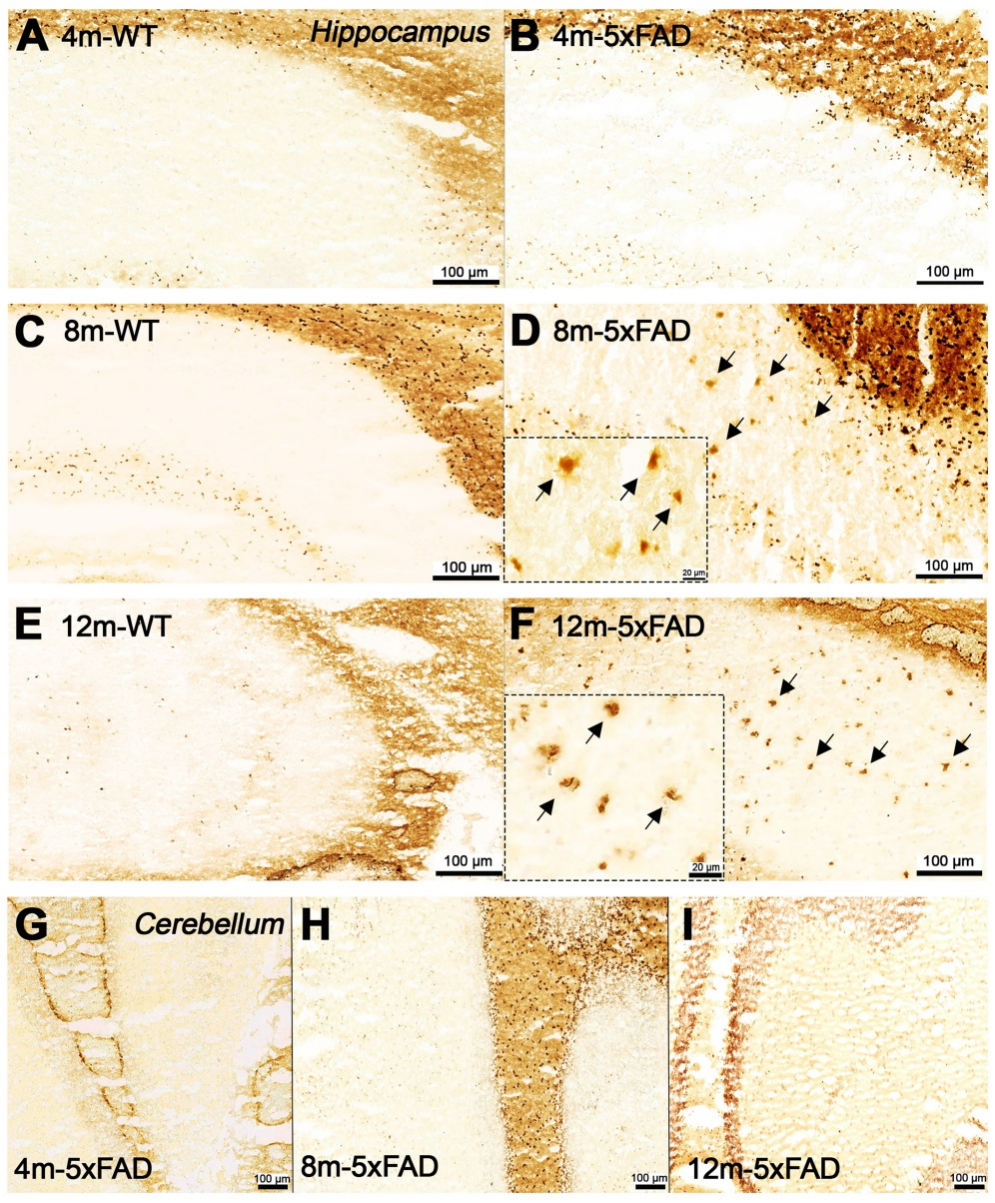
Staining for BChE enzymatic activity in 4, 8 and 12-month-old brains of WT (A, C and F) and 5xFAD mice (B, D, E and G-I) using the Karnovsky-Roots method. BChE staining showed increase of enzyme activity in the hippocampus of 5xFAD at different ages in comparison to WT (A to F) mice. Magnified images show the correspondence of plaques with BChE enzymatic activity in different regions of the cerebral cortex (B, D and E). Cerebellum showed low BChE staining in 4, 8 and 12-month-old 5xFAD mice (G-I).

Increased BChE enzymatic activity was observed in 5xFAD mice at the ages of 8 and 12 months with respect to 4 months in brain sub-regions investigated *ex vivo*, including the cerebral cortex (Figure 6), hippocampus (Figure 7A-F) and cerebellum (Figure 7 G-I). The white matter displayed high enzymatic activity of BChE, as shown by the corpus callosum, in both 5xFAD and WT mice. The BChE staining was completely diminished by the use of the BChE inhibitor ethopropazine in 12m-WT and partially inhibited in 5xFAD mice (Figure S3). We speculate that the use of low concentration of ethopropazine for inhibition (0.032 mM compared to 0.06 mM), used in other similar studies that inhibited BChE to visualize the AChE activity 30 or an obstruction of BChE binding site when bound to plaques could be a culprit for these results. Additional experiments will be conducted to validate these claims.

Qualitative comparison between BChE and Aβ abundance in brain slices of 12-month old 5xFAD mice suggest that the area covered by plaques, abundant with BChE, was smaller than that observed by Thioflavin S or 4G8 immunostaining. Though, general distribution of BChE corresponded well to the distribution of Aβ deposits, suggesting coexistence of the two biomarkers.

## Discussion

Compound **4** is a potent and selective BChE inhibitor, successfully used for restoration of cognitive abilities *in vivo* in mice. Previous studies demonstrated that compound **4** exhibits nanomolar affinity towards the active sites of BChE enzyme (K_i_ = 1.29 and 2.01 nM for - and + enantiomer, respectively) and that it works as an inhibitor of the enzyme activity (IC_50_ = 4.91 and 15.3 nM for - and + enantiomer, respectively) 16. Furthermore, compound **4** successfully helped mice recover from scopolamine-induced memory loss, indicating its function* in vivo*, probably through the inhibition of BChE 16. To obtain a broad profile of off-target activity for the compound **4** in CNS, *in vitro* safety pharmacology profiling (SafetyScreen44™ Panel) was conducted by Eurofins Discovery Poitiers, France, at 10 μM concentration of **4** (see full report for detailed experimental procedures and results in ESI-Annex). At this concentration, the compound showed no significant inhibition ability of dopamine (except for D_2S_ receptor; 55.6% inhibition, a value very close to the cut-off limit, set at 50%), cannabinoid, nicotinic, or opioid receptors (with the exception of µ-opioid receptor; 82.7% inhibition). The later, in a separate experiment, showed K_i_ value of 1.8 μM, as determined by displacement with tritiated [D-Ala^2^, N-Me-Phe^4^, Gly^5^-ol]-enkephalin ([^3^H]DAMGO) in rat cortex homogenates (see ESI for experimental details). The value was well above the K_i_ value for BChE inhibition, hence the competition, if any, would be insignificant. Compound **4** exhibited no significant inhibition of norepinephrine, dopamine or serotonin transporter proteins. Similarly, the BChE inhibitor showed no significant inhibition of cyclooxygenase, phosphodiesterase, monoamine oxidase-A, or AChE. The latter is in agreement with previously conducted *in vivo* safety experiments in mice that showed that the sulfonamide is not an agonist on cholinergic receptors, because it did not produce any acute cholinergic adverse effects, even at 100 mg/kg 16. On the other hand, the results showed inhibition values above cut-off limits, set at 50% inhibition, for muscarinic acetylcholine receptor (53.1%), adrenergic receptors α_1A_ and α_2A_ (71.7% and 60.9%, respectively) and for serotonin receptors (5-HT; a range from 84.3-96.2%). Additional experiments conducted in our lab showed only 48% and 78% displacement of [^3^H]flunitrazepam and [^3^H]8-OH-DPAT, respectively, from GABA_A_ and 5-HT_1A_ receptors at 0.3 mM, a concentration substantially higher than the ones intended to use in PET imaging (see ESI for experimental details). It was therefore concluded that the radioactive analogue of **4**, [^11^C]**4**, is an appropriate candidate for the use in *in vivo* determination of BChE abundance in the brain.

*Biodistribution and metabolism studies of [^11^C]**4** in WT mice showed favourable properties for in vivo PET imaging.* Radiotracers used for PET studies need to fulfil certain requirements. Besides high affinity for the target, PET tracers need to show sufficient blood circulation, fast clearance from non-target organs, easy penetration into the target tissue and low metabolism 34. In this work, *in vivo* PET studies were combined with *ex vivo* assays to assess biodistribution and metabolism of the newly developed PET tracer [^11^C]**4**. PET images showed rapid accumulation of radioactivity in the brain, with progressive decrease afterwards, demonstrating thus the capacity of [^11^C]**4** to cross the BBB, as anticipated from logP values, and the relatively fast wash-out. In order to have an estimation of blood clearance, the concentration of radioactivity in blood was estimated by drawing a VOI in the heart. No specific uptake was observed in the myocardium, and hence a VOI could not be drawn in the left ventricle, typical approach to obtain image-derived input functions 35. However, the delineation of a VOI in the heart provided valuable qualitative information that demonstrated a relatively fast blood clearance.

Extraction of blood samples showed the presence of two major metabolites of [^11^C]**4** at short times after administration. Noteworthy, these metabolites only partially accumulated in the brain (as derived from metabolite analysis in brain tissue) resulting in a higher fraction of parent compound in the brain when compared to that in the blood. Although not investigated in this study, the plasma metabolism analysis using LC-HRMS in previous work reports the detection of three possible metabolites: *N*-debenzylated, demethylated, and a demethylated + glucoronidated products 16. Indeed, our radio-HPLC study revealed the presence of metabolites with higher polarities (t_R_ = 2.0 and 3.4 min), which is consistent with the proposed metabolic pathway in the above-mentioned study. The major radioactive peak (t_R_ = 2 min) could therefore correspond to [^11^C]methanol, a side product of demethylated metabolite (compound **3**, see Scheme 1), and the minor radioactive peak (t_R_ = 3.4 min) to [^11^C]*N*-debenzylated metabolic product. While [^11^C]*N*-debenzylated metabolite presented only 6% overall radioactivity in the brain at 15 min after injection, non-radioactive compound **3** exhibits 10-fold lower inhibitory potency towards BChE than the parent compound (**3**, IC_50_ = 48.48 ± 7.48 nM, see ESI for more information). This, together with the relatively high molar activity of the radiotracer (which guarantees a very low molar concentration), suggests that the non-radioactive metabolite **3** shows negligible competition with [^11^C]**4** for binding to BChE. Altogether, our results confirm that [^11^C]**4** has favourable distribution and metabolism properties to assess BChE abundance in the brain using *in vivo* PET imaging.

*Blocking studies confirmed that [^11^C]**4** specifically binds to the chosen target both ex vivo and in vivo.* BChE blocking studies performed on brain tissue slices for both WT and AD animals using compound **4** and ethopropazine hydrochlorides as the blockers showed a decrease in the concentration of radioactivity in the presence of the blocker. Significant differences between AD and WT animals were found for ethopropazine at all investigated Bregma values (Figure S1), while in the case of **4,** differences were only significant at Bregma = -2.8. Noteworthy, higher radioactivity concentration values were also observed for AD animals at Bregma = 1.2 and -5.8, with P values of 0.156 and 0.075. The lack of significant differences in these cases can be attributed to two reasons. First, images were acquired with a PET camera, due to the unavailability of an autoradiography system, severely limiting spatial resolution and sensitivity; and second, very low amount of radioactivity was used during the incubation, in order to maintain the concentration of [^11^C]**4** below the K_i_ values. Still, differences could be observed, although the low values suggest that the density of BChE (B_max_) in the brain is low.

Encouraged by the positive results, we tackled *in vivo* studies using the hydrochloride salt of the parent compound **4**. Our results showed an initial increase of the radioactivity in the brain in both WT and AD animals, as demonstrated by PET imaging (Figures 2A and 2B). Although it seems surprising at first, the addition of a high concentration of hydrochloride salt of non-labelled compound **4** can displace [^11^C]**4** bound to BChE binding sites in tissues and blood, which results in an increase of the amount of free radiotracer in plasma and the consequent increase in total uptake of the radiotracer in the brain 36. Noteworthy, [^11^C]**4** was gradually washed out of the brain, resulting in lower abundance of the compound at the end of the imaging protocol in BChE-blocked animals compared to the control, non-BChE-blocked group for AD animals. Contrarily, similar levels of radiotracer uptake were achieved for WT animals in the presence and absence of the blocker (Figure 2B). These results, altogether, confirmed that [^11^C]**4** specifically binds to BChE and that the brain uptake of [^11^C]**4** can be used as a surrogate for BChE abundance *in vivo*.

*Longitudinal PET studies show progressive accumulation of [^11^C]**4** in 5xFAD mouse model for AD in the first 8 months.* The average uptake of the radiotracer in different brain sub-regions suggested elevated BChE in the brain of the mouse model for AD compared to the WT littermates in an age-dependant manner. At the whole brain level, SUV mean values for AD animals obtained when analysing the last time frame of dynamic time-activity curves (*ca.* 50-60 min after tracer administration) exhibited an increment of 7.5% and 12.2% for 6 and 8 months with respect to 4 months. These values are consistent with *ex vivo* and *in vivo* blocking experiments, which showed values close to 10% and 20% decrease in uptake under blocking conditions, respectively. Of note, decrement of radioactive signal in the brains observed in the *in vivo* blocking studies is slightly larger than the increase of the signal uptake, observed in 5xFAD mice between 4 and 8 months of age. Although there are many possible reasons behind this discrepancy, including the differences in experimental set-up and spatial-resolution limitations of equipment (note that both experiments were carried out with different PET systems), off-target binding could be one possible reason for these differences. The reasons behind this phenomenon will be addressed in a new study in more details on larger animal models.

Increased uptake, observed in different brain regions, including cerebral cortex, hippocampus, striatum, brain stem, cerebellum and thalamus, was in agreement with a previous work in which BChE presence was investigated *in vivo* using SPECT 18, and prevented the selection of an appropriate reference brain region, that would enable representation of the results as SUV relative to a common unaffected brain region.

Following a steady increase of the uptake of [^11^C]**4** in AD mice during the disease progression (4-8 months of age; Figures 3 and 4) the uptake decreased at 10 and 12 months of age. Indeed, at 12 months of age SUV values for all investigated brain regions were lower than those measured at the age of 4 months, and slightly lower than those obtained for WT age-matched littermates. However, the presence of BChE in the different AD-brain regions could still be observed in *ex vivo* staining studies (*vide infra*). The reasons behind this unexpected phenomenon remain unclear. One explanation for the low brain uptake of [^11^C]**4** in AD aged animals can be found in the binding mode of compound **4**. As previously reported 16, **4** interacts with Tyr332 side chain of human BChE *via* the positively charged nitrogen atom in the piperidine moiety, while the benzyl ring fits into the choline-binding pocket (groove contributed by Tyr82, Tyr332, Trp430, and Tyr440) and the naphthalene moiety interacts with Trp231 in the acyl-binding pocket via π-π interaction. Additionally, one of the sulfonamide oxygen atoms forms H-bond to the hydroxyl oxygen of Thr120. Binding of Aβ to the alleged “P-site - peripheral binding site” of BChE might limit the binding of compound **4** to BChE. Although the interaction of AChE and Aβ was demonstrated on several occasions 37, 38, for BChE the proof of binding is more elusive 39. In this study high levels of mature β-amyloid plaques at advanced stages of the disease (IHC analysis) were found in brain regions that exhibited diminished uptake of [^11^C]**4**, while the drop in the uptake of [^11^C]**4** was less severe in areas with low Aβ accumulation, i.e. the brain stem. Nevertheless, this is only a speculation and further experiments are needed to support this theory. Other factors, such as brain deterioration and decreased blood flow/perfusion, previously observed in a different mouse model of AD 40, could also contribute to this phenomenon.

*Longitudinal PET-[^18^F]florbetaben imaging is capable of in vivo detection of Aβ in 5xFAD animal model. The trend of increase of Aβ levels corresponds to BChE abundance from 4 to 8 months of age.* This is the first study to evaluate longitudinal amyloid-PET evolution using [^18^F]florbetaben in 5xFAD AD model. Our results showed a significant increase of [^18^F]florbetaben uptake in the cortex and the hippocampus in months 5-11, which is consistent with Aβ deposition, previously reported for this animal model 41. In addition, these results are in agreement with those described by Brendel and colleagues who showed a progressive [^18^F]florbetaben uptake increase from months 5 to 20 in different transgenic AD mouse models 42. The increase in the presence of Aβ in the cortex and the hippocampus is in good agreement with the increased uptake of [^11^C]**4** in these regions. However, there was a mismatch in white matter-rich tissues, i.e. the brain stem and the cerebellum. While no Aβ plaques deposition was observed in the cerebellum (used as the reference region) and the brain stem of 5xFAD mice (Figure 5), BChE abundance followed the trend observed in other brain regions.

*Post mortem IHC results partially correlate with in vivo findings.* Firstly, a substantial increase in BChE levels throughout the brain of 5xFAD, but not WT control, mice from 4 to 8 months of age was in a good agreement with PET imaging data. Contrarily to *in vivo* results, the *ex vivo* evaluation of 5xFAD mice brains showed a substantial accumulation and deposition of BChE at 12 months of age, while total and fibrillar Aβ in plaques were also increased in both the cortex and hippocampus of 5xFAD mice at the age 12 months, corroborating the results observed with PET imaging. No difference in Aβ plaques deposition was observed in the cerebellum between AD and WT mice, confirming that this brain region is appropriate as reference region in this mouse model. Contrary to our expectations, *ex vivo* analysis showed minimal accumulation of BChE in the cerebellum of 5xFAD mice. This was surprising, especially because PET imaging and blocking studies confirmed the presence of BChE in this brain region. The observed increase in [^11^C]**4** uptake in the white matter-rich areas, such as cerebellum, is expected if one considers that the increased demand for BChE is provoked by amyloid-associated damage. In fact, there is a large pool of evidence that suggest that BChE is present throughout the brain, mostly in subcortical layers and the white matter 43, 44. Furthermore, the increase of BChE enzymatic activity in the brains of AD patients, especially in the white matter and glia cells, has previously been observed 45. On the other hand, the result from *ex vivo* studies could be attributed to some compromises that needed to be made when analysing the stained tissue. Because all of the tissue sections were examined under the same conditions, to enable qualitative comparison between BChE deposition in different brain regions, high signal emanating from accumulated BChE around the plaques could overshadow the signal from dispersed BChE, rendering visualization of the enzyme in the cerebellum. Indeed, regional distribution of Aβ and BChE *ex vivo* revealed that Aβ deposited in areas with increased BChE abundance. This suggests coexistence of these two biomarkers and supports the theory that Aβ-driven conformational changes could lead to diminished binding capacity of [^11^C]**4**. Although further in-depth study of the effect of conformational changes of Aβ/BChE complexes on the binding affinity of **4** has not been conducted, the existence of such complexes has already been described in the literature 46, 47. Considering these findings, significant drop in [^11^C]**4** uptake at late stages of the disease in 5xFAD mice is not that surprising. Even more, substrates, used to assess BChE activity in the past 20 showed similar results.

In conclusion, compound **4** is a potent inhibitor of BChE that, when appropriately radiolabelled, can be used for PET-imaging of AD progression in a mouse model for AD. Longitudinal PET imaging study on 5xFAD mice and its age-matched WT littermates, with the same genetic background, using carbon-11 radiolabelled [^11^C]**4**, revealed that the trend of longitudinal increase of BChE levels in the brain between AD and control mice correspond to Aβ deposition rate from 4 to 8 months of age. This was further confirmed by *ex vivo* BChE staining. Furthermore, this study reveals the importance of longitudinal evaluation of disease animal models and opens new insights into the disease progression from the perspective of BChE enzyme. It shows the importance of appropriate data evaluation and critical view of the results, especially when *in vivo* and *ex vivo* data does not match. This said, the use of larger animal models and human tissue is currently in progress to confirm our findings, to dig deeper into the mechanism of the disease, and to support the discovery of new diagnostic and therapeutic tools for AD. This study is a first step towards the use of BChE as a possible biomarker for the early or differential diagnosis of AD.

## Figures and Tables

**Figure 8:**
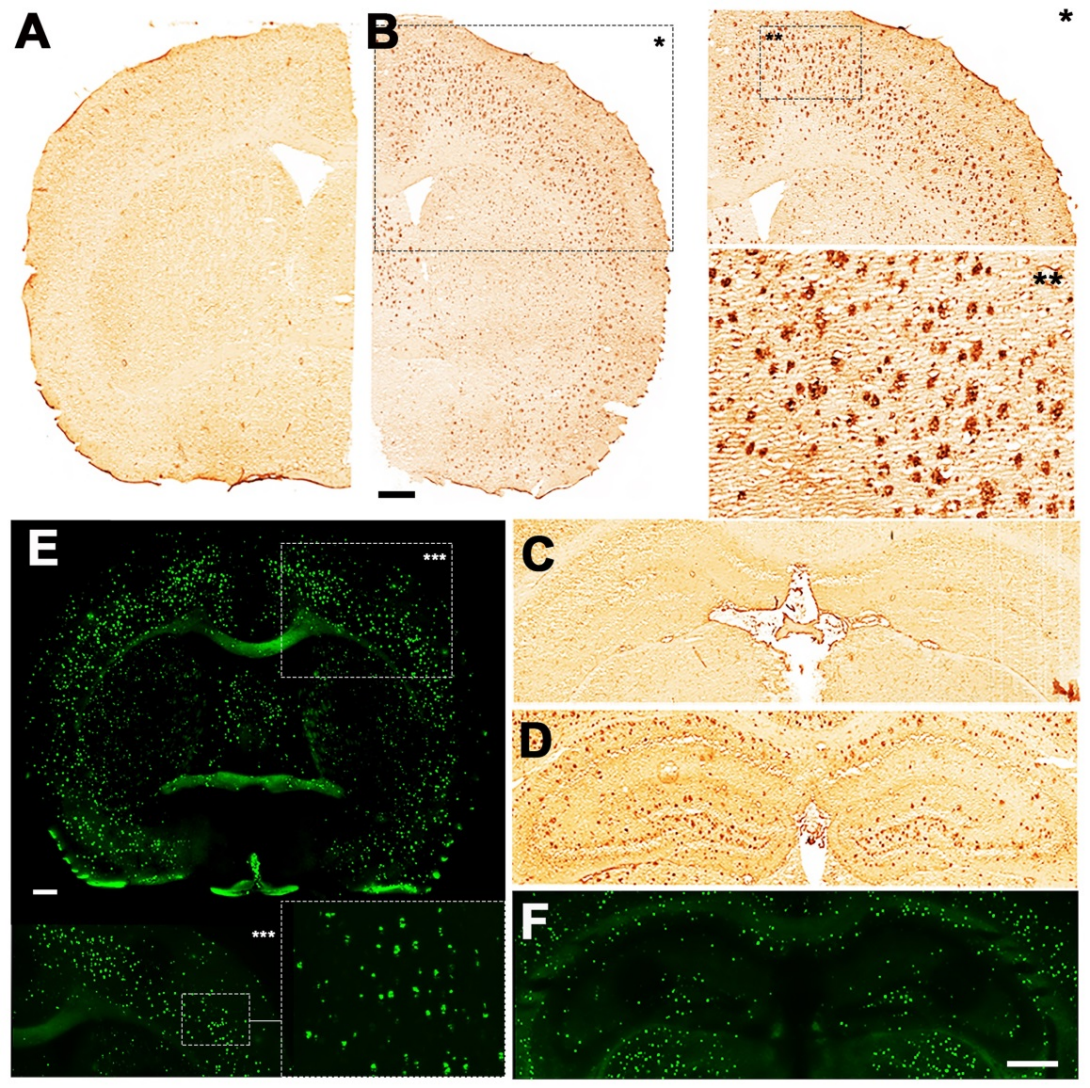
Deposition of Aβ in 12-month-old brains of WT (A and C) and 5xFAD mice (B, D, E, F). 4G8 antibody showed increase of Aβ plaque deposition in both the cortex (B) and hippocampus of 5xFAD (D) but not in WT mice (A and C). Magnified pictures of the cortex reveal a detailed distribution of Aβ plaques (B). Thioflavin S showed fibrillar amyloid accumulation in both the cortex (E) and hippocampus (F) of 5xFAD mouse. Scale bars, 0.5 mm.

## Abbreviations

Aβ: amyloid beta
ACh: acetylcholine
AChE: acetylcholinesterase
AD: Alzheimer disease
AMBMC: Australian Mouse Brain Mapping Consortium
BBB: blood brain barrier
BChE: butyrylcholinesterase
BS: brain stem
CC: corpus callosum
ChEs: cholinesterases
CP: cerebellar peduncle
CT: computerized tomography
CTX: cortex
DAMGO: [D-Ala^2^, N-Me-Phe^4^, Gly^5^-ol]-enkephalin
DMF: dimethylformamide
DTI: diffusion tensor imaging
FA: fractional anisotropy
FBP: filtered back projection
FOV: field of view
HIP: hippocampus
HPLC: high performance liquid chromatography
LC-MS: liquid chromatography-mass spectrometry
LFB: Luxol fast blue
MR: magnetic resonance
MRI: magnetic resonance imaging
MS: mass spectroscopy
PBS: phosphate buffer saline
PET: positron emission tomography
ROI: region of interest
SD: standard deviation
SPECT: single photon emission computerized tomography
SUV: standard uptake value
SUVr: relative standard uptake value
T_2_W: T_2_-weighed
TAC: time activity curve
TFA: trifluoroacetic acid
TR/TE: repetition time/echo time
VOI: volume of interest
WT: wild type
