# Supplementary material for: Longitudinal evaluation of a novel BChE PET tracer as an early in vivo biomarker in the brain of a mouse model for Alzheimer disease
Source: Theranostics. 2021 Apr 26;11(13):6542–59. doi: 10.7150/thno.54589 (PMC8120209; doi:10.7150/thno.54589)
Supplement: Supplementary file 1 — Supplementary figures and tables. [file thnov11p6542s1.pdf]

Longitudinal evaluation of a novel BChE PET tracer as an early *in vivo* biomarker in the brain of a mouse model for Alzheimer disease

Luka Rejc,[a,*] Vanessa Gómez-Vallejo,[b] Ana Joya,[b,c] Ander Egimendia,[b,d] Pilar Castellnou,[b] Xabier Ríos-Anglada,[b] Unai Cossío,[b] Zuriñe Baz,[b] Rossana Passannante,[b] Ignacio Tobalina-Larrea,[e,f] Pedro Ramos-Cabrer,[b,g] Albert Giralt,[h,i,j,k] Magdalena Sastre,[l] Estibaliz Capetillo-Zarate,[g,j,m] Urban Košak,[n] Damijan Knez,[n] Stanislav Gobec,[n] Mariel Marder,[o] Abraham Martin,[c,g] Jordi Llop[b,p,†]

[a] *University of Ljubljana, Faculty of Chemistry and Chemical Technology, Večna pot 113, 1000 Ljubljana, Slovenia*

[b] *CIC biomaGUNE, Basque Research and Technology Alliance (BRTA), Paseo Miramon 182, 20014, San Sebastian, Spain.*

[c] *Laboratory of Neuroimaging and biomarkers of inflammation, Achucarro Basque Center for Neuroscience, Science Park UPV/EHU, Sede building B. Sarriena, 48940 Leioa, Spain*

[d] *Multiple Sclerosis Unit, Biodonostia Health Institute, Donostia-San Sebastián, Spain*

[e] *Department of Nuclear Medicine, University Hospital of Araba (HUA), 01009 Vitoria-Gasteiz, Spain.*

[f] *Department of Surgery Radiology and Physical Medicine, Faculty of Medicine, University of the Basque Country, UPV/EHU,01009 Vitoria-Gasteiz, Spain*

[g] *IKERBASQUE, Basque Foundation for Science, 48013 Bilbao, Spain*

[h] *Departament de Biomedicina, Facultat de Medicina i Ciències de la Salut, Institut de Neurociències. Barcelona, Spain.*

[i] *Institut d'Investigacions Biomèdiques August Pi i Sunyer (IDIBAPS), 08036 Barcelona, Spain.*

[j] *Centro de Investigación Biomédica en Red sobre Enfermedades Neurodegenerativas (CIBERNED), 28031 Madrid, Spain.*

[k] *Production and Validation Center of Advanced Therapies (Creatio), Faculty of Medicine and Health Science, University of Barcelona, 08036 Barcelona, Spain.*

[l] *Department of Brain Sciences, Imperial College London, Hammersmith Hospital, Du Cane Road, London W12 0NN.*

[m] *Department of Neuroscience, Faculty of Medicine and Nursery, University of the Basque Country UPV/EHU and Achucarro Basque Center for Neuroscience, Barrio Sarriena S/N, 48940 Leioa, Spain.*

[n] University of Ljubljana, Faculty of Pharmacy, Aškerčeva cesta 7, 1000 Ljubljana, Slovenia.

[o] Universidad de Buenos Aires, Consejo Nacional de Investigaciones Científicas y Técnicas, and Instituto de Química y Fisicoquímica Biológicas, Facultad de Farmacia y Bioquímica, Universidad de Buenos Aires, Junín 956, C1113AAD Buenos Aires, Argentina

[p] Centro de Investigación Biomédica en Red – Enfermedades Respiratorias (CIBERES).

Corresponding authors

Luka Rejc: luka.rejc@fkkt.uni-lj.si; +38614798596

Jordi Llop: jllop@cicbiomagune.esjllop; +34943005333

Reagents and general methods

Chemicals and solvents were obtained from commercial sources (i.e., Acros Organics, Alfa Aesar, Fluka, Merck, Sigma-Aldrich, TCI Europe) and were used without purification unless otherwise stated. Ultrapure water (resistivity > 18MΩcm) was generated using a Milli-Q system (Millipore, Bedford, MA, USA).

Chemical reactions were monitored using analytical thin-layer chromatography plates (Merck 60 F254, 0.20 mm), and the components were visualized with ultraviolet light and/or through staining with ninhydrin reagent solution. Evaporation of solvents was performed at reduced pressure. Flash column chromatography was performed on silica gel 60 (0.040–0.063 mm) for column chromatography (particle size, 230–400 mesh). ^{1}H NMR and ^{13}C NMR were recorded on a Bruker Avance III NMR 400 MHz spectrophotometer at 400.130 and 100.613 MHz, respectively, and 295 K, unless otherwise stated. The chemical shifts (δ) are reported in *parts per million* (*ppm*) and are referenced to the deuterated solvent used. The coupling constants (*J*) are reported in Hz, and the splitting patterns are indicated as bs (broad singlet), bd (broad doublete), dd (doublet of doublets), t (triplet), and m (multiplet). Infrared spectra were obtained on a PerkinElmer FT-IR System Spectrum BX or on a Thermo Nicolet. High resolution mass spectra were recorded on a Q Executive Plus LC-MS/MS system (Thermo Scientific, MA, USA).

Analytical reversed phase method A was performed using an Agilent 1200 Series system equipped with a radioactivity detector (Gabi, Raytest) and a variable wavelength detector (λ = 350 nm) connected in series. A RP-C18 column (Mediterranea Sea18, 150 × 4.6 mm, 5 µm; Teknokroma, Spain) was used as the stationary phase and ascorbate buffer solution (20 g of ascorbic acid + 4.54 g NaOH in 2 L water, pH adjusted to 8.7 with 0.1 M NaOH; this solution diluted 1:1 with water)/acetonitrile (40/60, v/v) as the mobile phase. The sample solution (20 µL) was injected and eluted at a flow rate of 1 mL/min.

Analytical reversed phase HPLC method B was performed on an LC system (Dionex Ultimate 3000 Binary Rapid Separation; Thermo Scientific) equipped with an auto-sampler, a binary pump system, a photodiode array detector (λ set to 210, 254, and 280 nm), a thermostated column compartment, and

the Chromeleon chromatography data system. A Zorbax Eclipse Plus (150 mm × 4.6 mm, 5 µm; Agilent) protected with an HPLC guard cartridge system (octadecyl; 4.0 mm × 3.0 mm ID; Security Guard Cartridge C18 CODS, Phenomenex) was used as the stationary phase. The HPLC column was thermostated at 30 °C. The sample solution (10 µL, 0.1 mg/mL in MeOH) was injected and eluted at a flow rate of 1 mL/min by using a linear gradient of mobile phase A (0.10 % [v/v] aqueous TFA) and mobile phase B (MeOH). The gradient for mobile phase B was: 0−12 min, 10%−90%.

Analytical reversed phase method C was performed using the same chromatographic system and stationary phase as in method A. As the mobile phase, 0.1% [v/v] TFA in water (A)/acetonitrile (B) was used, with the following gradient: t = 0 min, 90% A; t = 1 min, 90% A; t = 10 min, 25% A; t = 13min, 25% A; t = 14 min, 90% A; t = 15 min, 90% A. The sample solution (20 µL) was injected and eluted at a flow rate of 1.5 mL/min.

Chemistry and radiochemistry

Production of [^{18}F]florbetaben

The synthesis of [^{18}F]florbetaben was performed using a TRACERlab FX_{FN} synthesis module (GE Healthcare) by ^{18}F-fluorination of the *N*-BOC-protected precursor followed by hydrolysis, as previously described [1] with minor modifications. In brief, [^{18}F]F^{-} was first trapped on a pre-conditioned Sep-Pak® Accell Plus QMA Light cartridge (Waters, Milford, MA, USA), and then eluted with a solution of Kryptofix $K_{2.2.2}$/K_2CO_3 in a mixture of water and acetonitrile. After complete elimination of the solvent by azeotropic evaporation, a solution containing the precursor (3 mg) in dimethylsulfoxide (1 mL) was added and the mixture was heated at 165 °C for 5 min. The reactor was then cooled at room temperature, 10% HCl aqueous solution was added (0.25 mL) and the mixture was heated (2.5 min, 90 °C). The reaction crude was then diluted with NaOH solution (0.33 mL, 0.1 g/mL) and 3 mL of mobile phase, and purified by HPLC using a Nucleosil 100-7 C18 column (Macherey-Nagel, Düren, Germany) as stationary phase and aqueous ascorbate buffer solution (20 g of ascorbic acid + 4.54 g NaOH in 2 L water, pH adjusted to 8.7 with 0.1 M NaOH; this solution diluted 1:1 with water)/acetonitrile (40/60, v/v) as the mobile phase at a flow rate of 5 mL/min. The desired fraction (t_R = 29–30 min) was collected, diluted with water (20 mL), and the radiotracer was

retained on a C-18 cartridge (Sep-Pak® Light, Waters, Milford, MA, USA) and further eluted with ethanol (1 mL) and ascorbate buffer solution (20 g of ascorbic acid + 4.54 g NaOH in 2 L water, pH adjusted to 8.7 with 0.1 M NaOH; 5 mL). Filtration through a 0.22 µm filter yielded the final solution, ready for injection. Chemical and radiochemical purity and molar activity were determined by HPLC using method A (t_R = 6.0 min).

Production of (±)[^{11}C]*N*-((1-Benzylpiperidin-3-yl)methyl)-*N*-(2-methoxyethyl)naphthalene-2-sulfonamide ([^{11}C]**4**)

The synthesis of the appropriate precursor for the preparation of (±)[^{11}C]*N*-((1-Benzylpiperidin-3-yl)methyl)-*N*-(2-methoxyethyl)naphthalene-2-sulfonamide ([^{11}C]**4**) was carried out following the synthetic route shown in Scheme 1A.

A

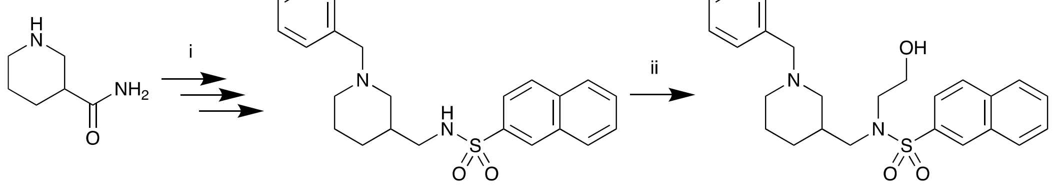

B

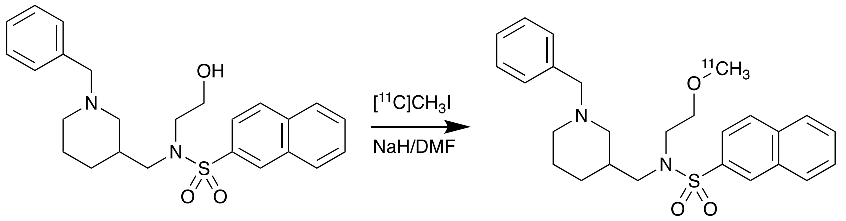

Scheme 1. A) Reaction for the preparation of (±)*N*-[(1-benzylpiperidin-3-yl)methyl]-*N*-(2-hydroxyethyl)naphthalene-2-sulfonamide (**3**). Reagents and conditions: (i) see ref. [2]; (ii) 2-bromoethane-1-ol, K_2CO_3, dry DMF, room temperature to 100 °C, 18 h, under argon; B) radiosynthesis of [^{11}C]**4** by ^{11}C-methylation of the precursor **3**.

First, sulfonamide **2** was synthesized from nipecotamide **1** as previously described [2]. Sulfonamide **2** was then reacted with 2-bromoethan-1-ol in the presence of K_2CO_3 in dry dimethylformamide (DMF) at 100 °C to produce racemic alcohol **3**. In brief: a dry round bottom 100-mL flask equipped with a stirring bar was charged with sulfonamide **2** (2.074 g, 5.257 mmol, 1.0 equiv.) under argon. Dry DMF

(50 mL) was added via a double tipped needle. The solution was stirred and K_2CO_3 (2.180 g, 15.774 mmol, 3.0 equiv.) was added. 2-Bromoethan-1-ol (1.118 mL, 15.774 mmol, 3.0 equiv.) was added to the resulting suspension and the reaction mixture was then stirred at 100 °C for 18 h. The mixture was cooled to room temperature and evaporated. CH_2Cl_2 (100 mL) was added to the residue and the resulting suspension was filtered into a 250-mL separating funnel and washed with water (3 × 100 mL). The organic phase was dried with Na_2SO_4, the suspension filtered and the filtrate evaporated. The residue was purified by flash column chromatography using CH_2Cl_2/MeOH (20:1, v/v) as the eluent, to produce 1.962 g of (±)*N*-[(1-benzylpiperidin-3-yl)methyl]-*N*-(2-hydroxyethyl)naphthalene-2-sulfonamide **3** as a colorless oil which solidified into a white solid after cooling (85% yield). R_f = 0.42 (CH_2Cl_2/MeOH, 10:1, v/v). mp: 34–37 °C. IR (ATR): 3059, 2933, 1452, 1332, 1154, 1129, 1072, 859, 747, 651, 546 cm^{-1}. ^{1}H-NMR (400.130 MHz, $CDCl_3$): δ = 1.09–1.19 (m, 1 H), 1.45–1.55 (m, 1 H), 1.60–1.71 (m, 2 H), 2.14 (bs, 1 H), 2.26 (bs, 2 H), 2.46 (bs, 1 H), 2.61 (bd, *J* = 8.9 Hz, 1 H), 3.03–3.23 (m, 3 H), 3.33–3.39 (m, 1 H), 3.49 (dd, J_1 = 32.9 Hz, J_2 = 12.9 Hz, 2 H), 3.72 (t, *J* = 5.1 Hz, 2 H), 7.23–7.33 (m, 6 H), 7.60–7.68 (m, 2 H), 7.76 (dd, J_1 = 8.6 Hz, J_2 = 1.9 Hz, 1 H), 7.91–7.99 (m, 3 H), 8.37 (t, *J* = 0.9 Hz, 1 H). ^{13}C-NMR (100.613 MHz, $CDCl_3$): δ = 23.42, 27.52, 34.56, 52.11, 53.34, 53.64, 56.59, 61.71, 63.45, 122.47, 127.14, 127.51, 127.81, 128.10, 128.64, 128.72, 129.14, 129.34, 129.54, 132.05, 134.68, 135.17, 137.05. HRMS (ESI+): m/z calcd for $C_{25}H_{31}O_3N_2S$ 439.2050; found 439.2040. HPLC purity 99 %, at 254 nm (method B, t_R = 9.48 min).

The production of [^{11}C]**4** was carried out by ^{11}C-methylation of (±)*N*-[(1-benzylpiperidin-3-yl)methyl]-*N*-(2-hydroxyethyl)naphthalene-2-sulfonamide **3** (Scheme 1B). [^{11}C]CH_3I was produced in a TRACERlab $FX_{C\ Pro}$ synthesis module (GE Healthcare) using the gas phase method, as previously described [3], and distilled into a 2 mL stainless steel loop pre-charged with a solution containing the precursor (3 mg, 0.007 mmol) and NaH (1.7 mg, 0.05 mmol) in DMF (100 µL). After reaction (5 min, room temperature) the product was purified by semi-preparative HPLC using a RP-C18 column (Phenomenex, 250 × 10 cm, 5 µm particle size) as the stationary phase and 0.1% TFA in water/acetonitrile (55/45 v/v; flow rate = 5 mL/min) as the mobile phase. The product fraction (t_R =11 min) was collected, diluted with ultrapure water (20 mL) and circulated through a Sep-Pak C-18 Plus

cartridge (Waters) to trap [^{11}C]**4**. The cartridge was rinsed with purified water (5 mL) and the radiotracer was eluted with 1 mL of absolute ethanol and 4 mL of physiologic saline solution, which, after filtration through 0.22 µm filter, yielded the solution ready for use in *in vivo* studies. Chemical and radiochemical purity were determined by HPLC using method C (t_R = 9.8 min).

Determination of partition coefficient (log $P_{oct/wat}$) of [^{11}C]**4**

To a mixture of 5 mL 1-octanol and 5 mL ultrapure water was added 10 µL of [^{11}C]**4** (ca. 1.5 MBq) and shaken vigorously. The layers were let to separate and 3 samples (200 µL each) were collected for each phase and counted in an automatic gamma counter (2470 Wizard, PerkinElmer). Logarithm of partition coefficient was calculated as the logarithm of the amount of radioactivity contained in 1-octanol phase divided by the activity in ultrapure water.

Determination of cholinesterase inhibition of alcohol **3**

The inhibitory potencies of compound **3** against the cholinesterases (ChEs) were determined using the method of Ellman [4]. In brief, compound stock solutions in dimethylsulfoxide were incubated with Ellman's reagent and the ChEs in 0.1 M phosphate buffer, pH 8.0 (final concentrations: 370 µM Ellman's reagent; 1 nM or 50 pM human BChE or human AChE/murine AChE, respectively) for 5 min at room temperature. The reactions were started by addition of the substrate (butyrylthiocholine iodide/ acetylthiocholine iodide for BChE and AChE, respectively). The final concentration of the substrate and DMSO were 500 µM and 1% [v/v], respectively. The increase in absorbance at 412 nm was monitored for 1 min using a 96-well microplate reader (Synergy H4; BioTek Instruments, Inc., USA). The initial velocities in the presence (v_i) and absence (v_o) of the test compounds were calculated. The inhibitory potencies were expressed as the residual activities (RAs), according to Equation [1]:

$$RA = (v_i - b) / (v_o - b) \quad [1],$$

where b is the blank value using phosphate buffer instead of the ChEs. For IC_{50} determinations, seven different concentrations of each compound were used. The IC_{50} values were obtained by plotting the residual activities against the applied inhibitor concentrations, with the experimental data fitted to a four-parameter logistic function using GraphPad Prism 8.0 (GraphPad Software, CA, USA). Tacrine was used as the positive control. The K_i and IC_{50} values for compound **4** are taken from the reference

[2], where they were experimentaly determined according to the procedures decribed in the reference using the cold compound **4**.

***In vitro* binding assays**

For *in vitro* binding assays rat brain homogenates were used. Adult male Wistar rats (weight, 200–300 g) were obtained from the Central Animal House of the School of Pharmacy and Biochemistry, University of Buenos Aires, Buenos Aires, Argentina. The housing, handling, and experimental procedures for rats complied with the recommendations set out by the National Institutes of Health Guide for Care and Use of Laboratory Animals (NIH Publication No. 8023, revised 1996) and the Institutional Committees for the Care and Use of Laboratory Animals of the Faculty of Pharmacy and Biochemistry, University of Buenos Aires, Argentina (Code 31682/2014). Rats were humanely killed by decapitation and their brains were removed, washed and rapidly dissected on ice. Homogenization was performed using a homogenizer (PRO Scientific Inc) at 30,000 rpm for 1 min. The detailed description of the brain tissue homogenates preparations and the procedures for individual assay are described below. Protein concentration was determined by the method of Bradford using bovine serum albumin as standard. Compound **4** was dissolved in 10% DMSO in EtOH to prepare 30 mM stock solution. For active compounds in the receptor *in vitro* binding assays the dose-response curve was determined and IC_{50} and K_i values for compound binding were calculated. The IC_{50} value for radioligand displacement was obtained by plotting the residual activities against the applied concentration of **4**, with the experimental data fitted to a four-parameter logistic function using GraphPad Prism 6.0 (GraphPad Software, CA, USA). The K_d value for $[^3H]$DAMGO [L-Tyrosyl-3,5-^3H(*N*)-D-alanyl-glycyl-*N*-methyl-L-phenylalanyl-glycinol, a synthetic highly selective ligand for μ-opioid receptors] is 0.5 nM, and it was used for the calculation of the *Ki* value of compound **4** binding to the μ-opioid receptors according to the following equation: $K_i = IC_{50} / (1 + [X]/K_d)$, where X is the concentration of the $[^3H]$DAMGO used, and K_d is the dissociation constant of the $[^3H]$DAMGO.

$GABA_A$ receptor binding assay

The cortex tissues were homogenized in 10 volumes/weight of ice-cold 0.32 M sucrose and centrifuged at 900× *g* for 10 min at 4 °C. Supernatant was decanted and centrifuged at 100,000× *g* for

30 min at 4 °C. The pellet was washed twice with 25 mM Tris–HCl buffer (pH 7.4), and stored at −80 °C until use.

For the [^{3}H]flunitrazepam binding assay (the GABA$_A$ receptor binding assay), the membranes (protein concentration 1.93 mg/mL) were thawed and resuspended in 25 mM Tris–HCl buffer (pH 7.4), to a final protein concentration of 0.2 mg/mL. The incubation was carried out at 4 °C for 1 h in a final volume of 1 mL membrane suspension in the presence of **4** (300 µM) and with 0.33 nM [^{3}H]flunitrazepam. Nonspecific binding was determined in parallel incubations in the presence of 10 µM flunitrazepam. The incubation was terminated by dilution with 3 mL of 25 mM Tris–HCl buffer (pH 7.4), filtration under vacuum through Whatman GF/A glass fiber filters and two washes (3 mL each) of 25 mM Tris–HCl buffer (pH 7.4). Filters were counted after addition of Optiphase "Hisafe" 3 (Wallac, Turku, Finland) liquid scintillation cocktail.

5-HT$_{1A}$ receptor binding assay

For 5-HT$_{1A}$ binding assays, the cortex tissues were homogenized in 30 volumes/weight of ice-cold 50 mM Tris–HCl buffer (pH 7.4) and centrifuged at 40,000× *g* for 15 min at 0 °C to 4 °C. The resulting pellets were resuspended in 30 volumes/weight of the same buffer, and incubated at 37 °C for 20 min. The centrifugation step was repeated twice under the same conditions as described above, and the final pellets were resuspended in 30 volumes/weight of 50 mM Tris–HCl buffer (pH 7.4), and stored at –80 °C until use.

For the [^{3}H]-8-OH-DPAT binding assay (the 5-HT$_{1A}$ receptor binding assay), the membranes were thawed and resuspended in 50 mM Tris–HCl (pH 7.4) with 1 mM $MnCl_2$, to a final protein concentration of 0.5 mg/mL. The incubation was carried out at 25 °C for 1 h in a final volume of 1 mL membrane suspension in the presence of **4** (300 µM) and with 0.2 nM [^{3}H]-8-OH-DPAT (170.2 Ci/mmol). Serotonin was used as the positive control. Nonspecific binding was determined in parallel incubations in the presence of serotonin (10 µM). The incubation was terminated by dilution with 3.5 mL of 50 mM Tris–HCl buffer (pH 7.4), filtration under vacuum through Whatman GF/A glass fiber filters and two washes (3.5 mL each) of 50 mM Tris–HCl buffer (pH 7.4). Filters were counted after addition of Optiphase "Hisafe" 3 (Wallac, Turku, Finland) liquid scintillation cocktail.

µ-opioid receptor binding assay

For μ-opioid receptor binding assay, the forebrain tissues were washed with ice-cold 50 mM Tris–HCl (pH 7.4), homogenized in 10 volumes/weight of ice-cold 0.32 M sucrose and centrifuged at 900× *g* for 10 min at 4 °C. Supernatant was decanted and centrifuged at 100,000× *g* for 30 min at 4 °C. The resulting pellet was resuspended in 30 volumes/weight of 50 mM Tris–HCl (pH 7.4) and incubated at 37 °C for 30 min to remove any endogenous opioid peptides. The suspension was centrifuged at 100,000× *g* for 30 min at 4 °C, the pellet obtained was resuspended in 10 volumes/weight of 50 mM Tris–HCl (pH 7.4) and stored at –80 °C until use.

For the [^{3}H]DAMGO binding assay (the μ-opioid receptor binding assay), the membranes were thawed and resuspended in 50 mM Tris–HCl (pH 7.4) to a final protein concentration of 0.35 mg/mL. The incubation was carried out at 25 °C for 1 h in a final volume of 1 mL membrane suspension in the presence of **4** (300 μM) and with 0.86 nM of [^{3}H]DAMGO. Nonspecific binding was determined in parallel incubations in the presence of naltrexone (10 μM). The incubation was terminated by dilution with 3.5 mL of 50 mM Tris–HCl buffer (pH 7.4), filtration under vacuum through Whatman GF/A glass fiber filters and one washes (3.5 mL) of 50 mM Tris–HCl buffer (pH 7.4). Filters were counted after addition of Optiphase “Hisafe” 3 (Wallac, Turku, Finland) liquid scintillation cocktail.

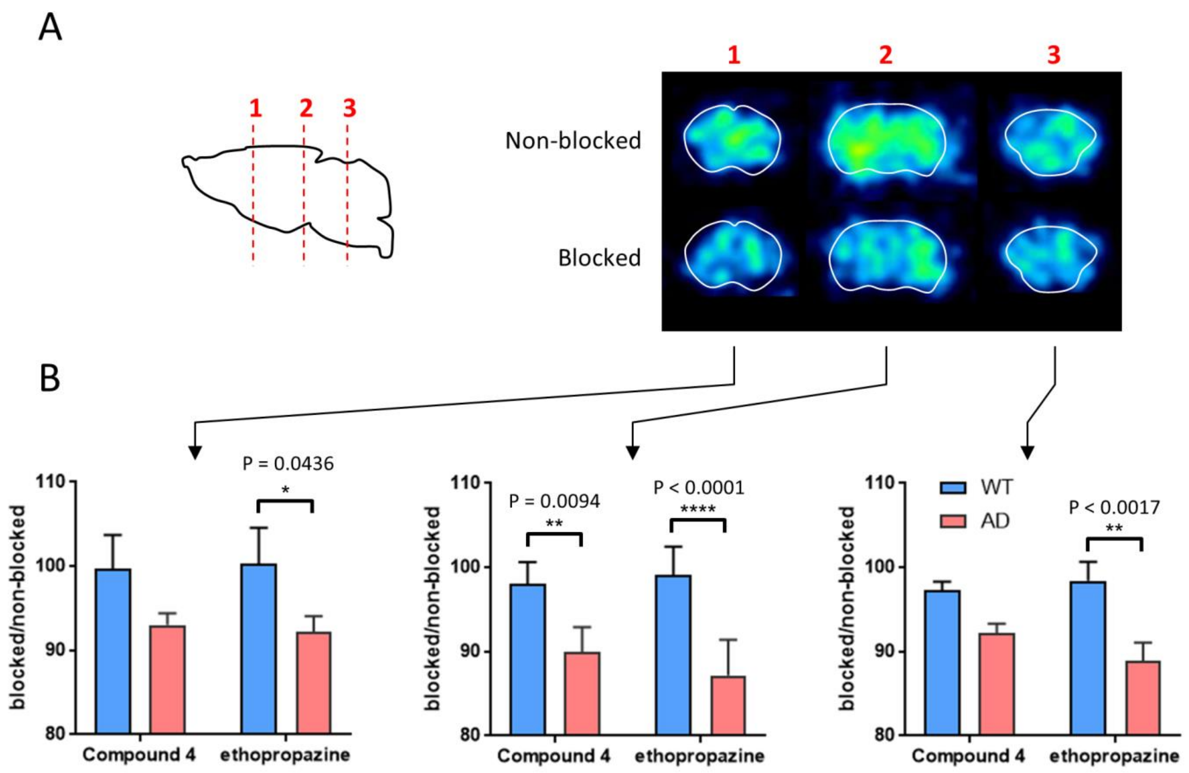

Figure S1. A) Left: representative drawing of a sagittal view of the mouse brain, with the Bregma positions 1.2 (**1**), -2.8 (**2**) and -5.8 (**3**); right: PET images obtained on brain tissue slices from AD animals corresponding to Bregma positions 1.2 (**1**), -2.8 (**2**) and -5.8 (**3**) after incubation with [^{11}C]**4** (top) and [^{11}C]**4** in the presence of compound **4** (200 nM; bottom); B) block-to-non-block ratios obtained on brain tissue slices corresponding to Bregma positions 1.2 (left), -2.8 (middle) and -5.8 (right) after incubation with [^{11}C]**4** (top) and [^{11}C]**4** in the presence of compound **4** or ethipropazine for WT (blue) and AD (red) animals. Probability values (difference AD vs. WT) are depicted as *(P < 0.05), ** (P < 0.01), *** (P<0.001) and **** (P<0.0001).

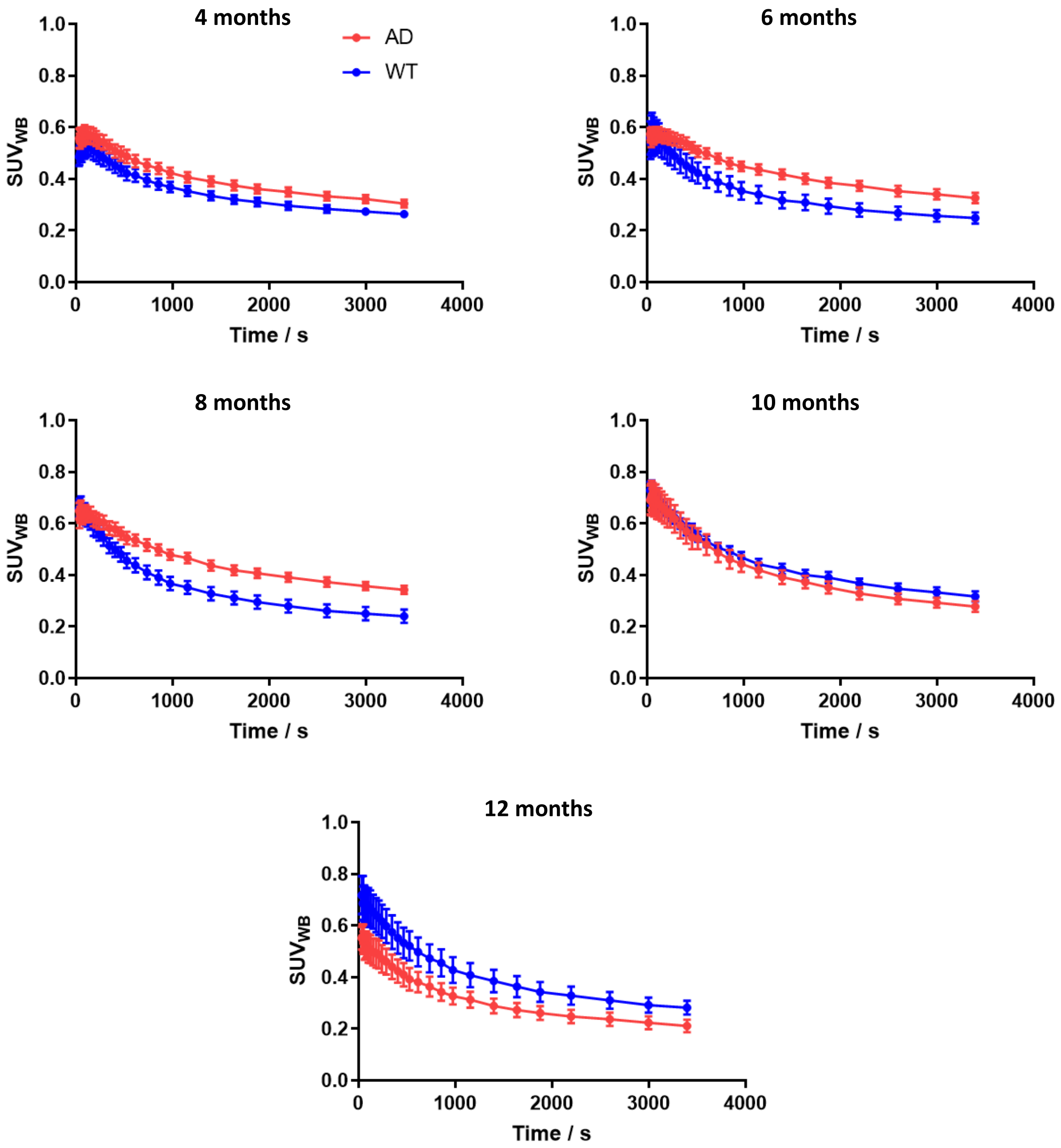

Figure S2. Time activity curves in the whole brain (WB; considered as the sum of all brain regions segmented in the T2 Mirrione brain template provided by π-MOD software) obtained from PET images after intravenous administration of $[^{11}C]$**4** to AD and WT animals at the ages of 4, 6, 8, 10 and 12 months.

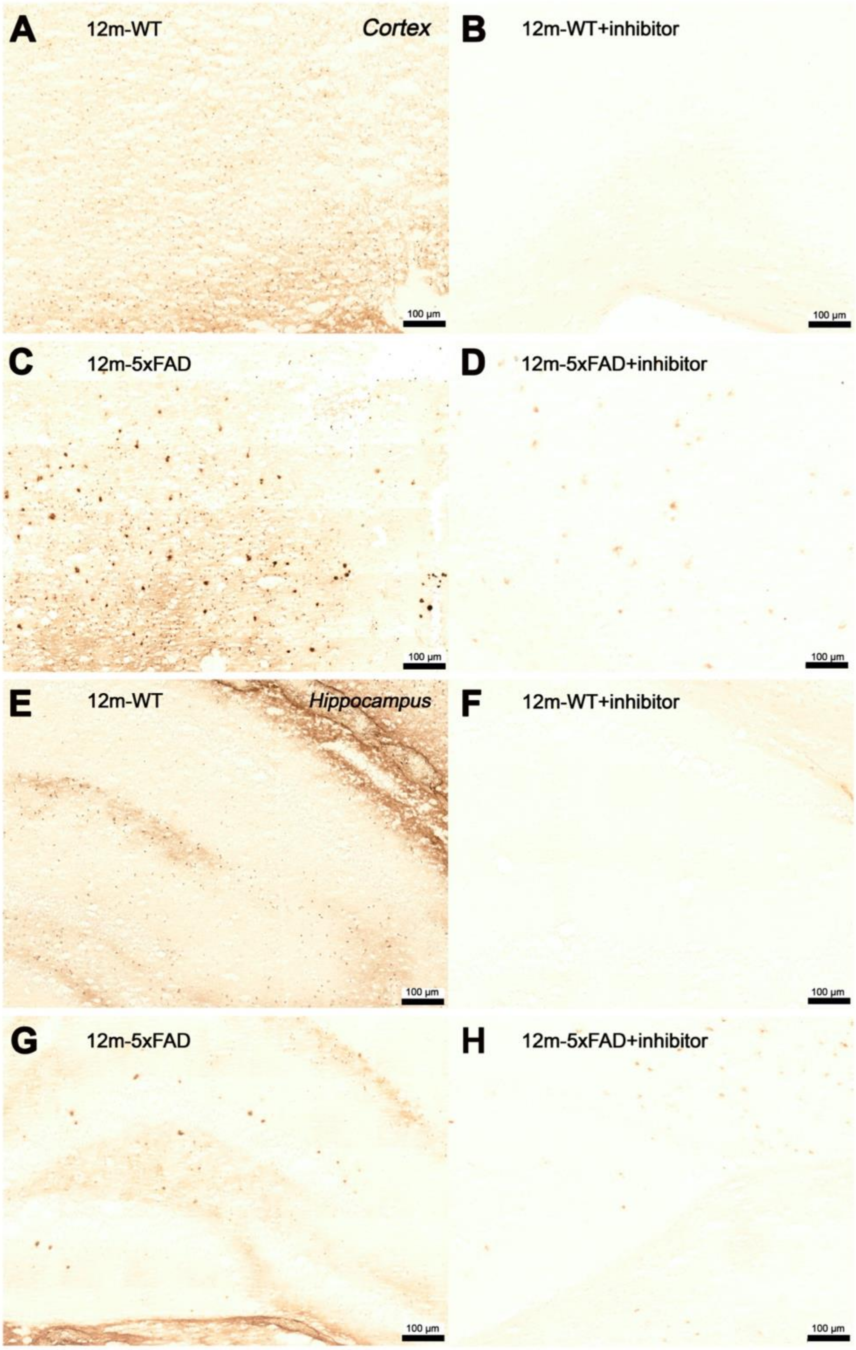

Fig S3. Staining for BChE enzymatic activity in 12-month-old brains of WT (A and E) and 5xFAD mice (C and G) using the Karnovsky-Roots method The use of the BChE inhibitor ethopropazine hydrochloride confirmed a decrease of BChE staining in both white matter of a 12m-WT mouse (B and F) and in plaques of cortex and hippocampus of a 12m-5xFAD mouse (D and H).

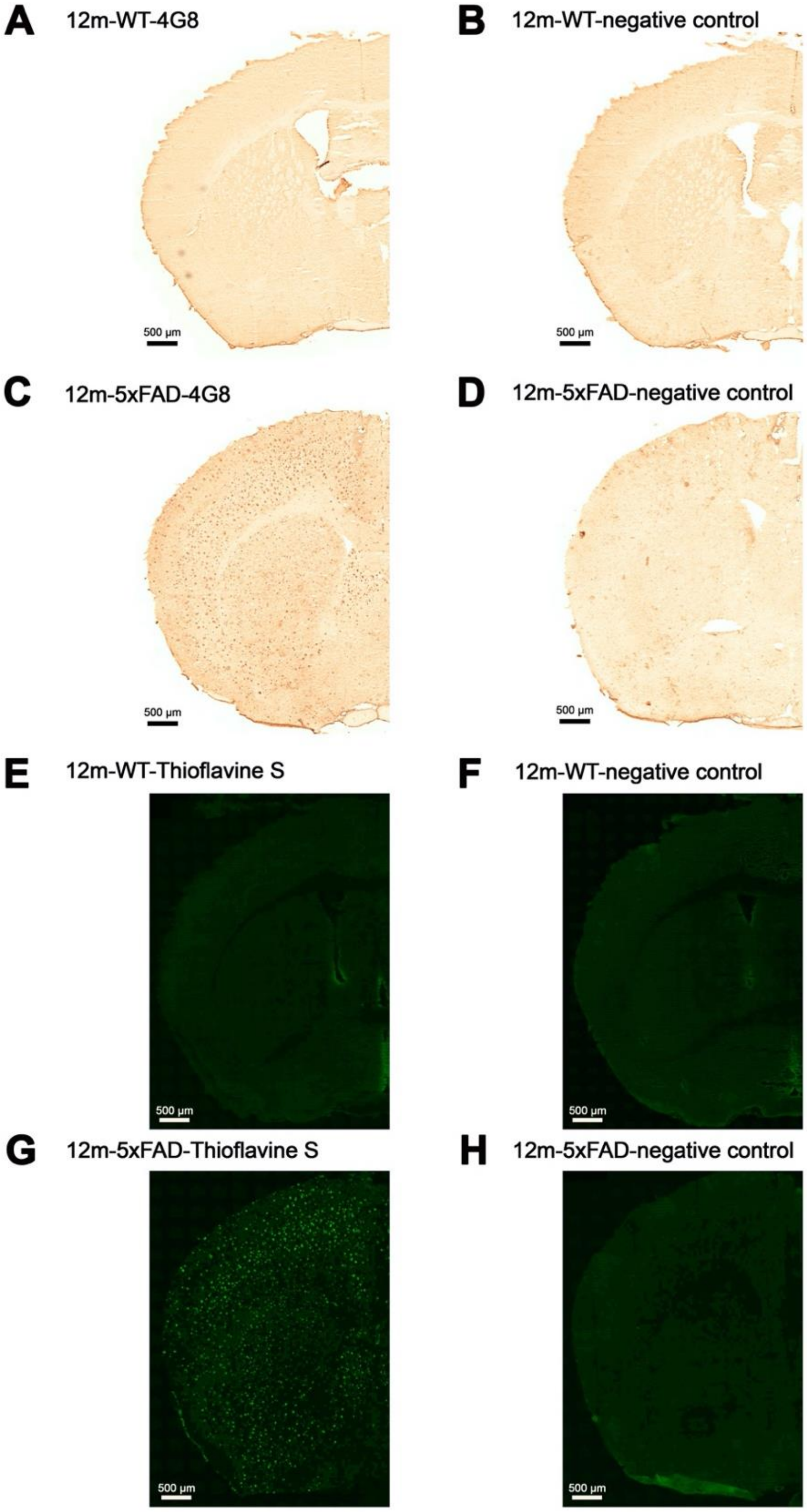

Fig S4. Staining for 4G8 antibody (A and C) and Thioflavine S (E and G) with the corresponding negative controls for 4G8 (B and D) and Thioflavine S (F and H) in the region of cerebral cortex and striatum of the brains of 12-month-old WT (A-B and E, F) and 5xFAD mice (C-D and G-H).

Table S1. Injected dose, molar activity (SA) and injected amount (IA) of [^{11}C]**4** in longitudinal, blocking and biodistribution PET studies. Note: Annotation N/A represents cases when the animal was not injected either because: (i) they have been sacrificed prior to the imaging session; (ii) tail damage which discouraged injection; (iii) sufficient number of scanned animals and limitation of access to PET camera; or (iv) unsuccessful injection, which resulted in unrepresentative data.

WT animals, longitudinal studies

4 months				
**Animal #**	**dose (µCi)**	**dose (MBq)**	**SA (GBq/µmol)**	**IA (nmol)**
1	240	8.9	175	0.051
2	304	11.2	170	0.066
3	416	15.4	155	0.099
4	218	8.1	212	0.038
5	405	15.0	178	0.084
6	377	13.9	187	0.075
7	307	11.4	215	0.053
8	319	11.8	145	0.081
9	232	8.6	167	0.051
10	253	9.4	256	0.037
Average	307.1	11.4	186.0	0.064
SDEV	72.6	2.7	33.1	0.021
Min	218.0	8.1	145.0	0.037
Max	416.0	15.4	256.0	0.099

6 months				
**Animal #**	**dose (µCi)**	**dose (MBq)**	**SA (GBq/µmol)**	**IA (nmol)**
1	39	1.4	121	0.012
2	141	5.2	146	0.036
3	211	7.8	150	0.052
4	155	5.7	132	0.043
5	157	5.8	120	0.048
6	244	9.0	146	0.062
7	173	6.4	121	0.053
8	273	10.1	155	0.065
9	339	12.5	145	0.087
10	N/A	N/A	N/A	N/A
Average	192.4	7.1	137.3	0.051
SDEV	86.7	3.2	13.9	0.021
Min	39.0	1.4	120.0	0.012
Max	339.0	12.5	155.0	0.087

8 months				
**Animal #**	**dose (µCi)**	**dose (MBq)**	**SA (GBq/µmol)**	**IA (nmol)**
1	164	6.1	185	0.033
2	393	14.5	112	0.130
3	252	9.3	155	0.060
4	73	2.7	164	0.016
5	82	3.0	146	0.021
6	284	10.5	175	0.060
7	N/A	N/A	N/A	N/A
8	119	4.4	161	0.027
9	172	6.4	172	0.037
10	262	9.7	188	0.052
Average	200.1	7.4	162.0	0.048
SDEV	105.8	3.9	23.1	0.034
Min	73.0	2.7	112.0	0.016
Max	393.0	14.5	188.0	0.130

10 months				
**Animal #**	**dose (µCi)**	**dose (MBq)**	**SA (GBq/µmol)**	**IA (nmol)**
1	266	9.8	147	0.067
2	274	10.1	118	0.086
3	174	6.4	198	0.033
4	134	5.0	186	0.027
5	235	8.7	175	0.050
6	239	8.8	185	0.048
7	355	13.1	144	0.091
8	244	9.0	212	0.043
9	N/A	N/A	N/A	N/A
10	N/A	N/A	N/A	N/A
Average	240.1	8.9	170.6	0.055
SDEV	66.2	2.4	31.5	0.024
Min	134.0	5.0	118.0	0.027
Max	355.0	13.1	212.0	0.091

12 months				
**Animal #**	**dose (µCi)**	**dose (MBq)**	**SA (GBq/µmol)**	**IA (nmol)**
1	N/A	N/A	N/A	N/A
2	325	12.0	201	0.060
3	354	13.1	198	0.066
4	374	13.8	188	0.074
5	416	15.4	182	0.085
6	N/A	N/A	N/A	N/A

7	303	11.2	212	0.053
8	327	12.1	183	0.066
9	188	7.0	194	0.036
10	N/A	N/A	N/A	N/A
Average	326.7	12.1	194.0	0.063
SDEV	71.6	2.6	10.7	0.016
Min	188.0	7.0	182.0	0.036
Max	416.0	15.4	212.0	0.085

AD animals, longitudinal studies

4 months				
**Animal #**	**dose (µCi)**	**dose (MBq)**	**SA (GBq/µmol)**	**IA (nmol)**
11	212	7.8	186	0.042
12	67	2.5	133	0.019
13	293	10.8	160	0.068
14	274	10.1	154	0.066
15	262	9.7	128	0.076
16	199	7.4	194	0.038
17	296	11.0	154	0.071
18	435	16.1	127	0.127
19	217	8.0	144	0.056
20	111	4.1	132	0.031
21	187	6.9	155	0.045
22	122	4.5	167	0.027
23	147	5.4	110	0.049
Average	217.1	8.0	149.5	0.055
SDEV	97.4	3.6	23.9	0.029
Min	67.0	4.1	110.0	0.027
Max	435.0	16.1	194.0	0.127

6 months				
**Animal #**	**dose (µCi)**	**dose (MBq)**	**SA (GBq/µmol)**	**IA (nmol)**
11	79	2.9	188	0.016
12	175	6.5	201	0.032
13	151	5.6	173	0.032
14	102	3.8	104	0.036
15	225	8.3	155	0.054
16	292	10.8	143	0.076
17	170	6.3	177	0.036
18	279	10.3	166	0.062
19	385	14.2	158	0.090
20	357	13.2	140	0.094
21	N/A	N/A	N/A	N/A

22	N/A	N/A	N/A	N/A
23	N/A	N/A	N/A	N/A
Average	221.5	8.2	160.5	0.053
SDEV	104.2	3.7	23.6	0.024
Min	79.0	3.8	104.0	0.036
Max	385.0	14.2	201.0	0.094

8 months				
**Animal #**	**dose (µCi)**	**dose (MBq)**	**SA (GBq/µmol)**	**IA (nmol)**
11	173	6.4	168	0.038
12	241	8.9	197	0.045
13	278	10.3	133	0.077
14	252	9.3	136	0.069
15	139	5.1	172	0.030
16	143	5.3	115	0.046
17	129	4.8	98	0.049
18	137	5.1	121	0.042
19	N/A	N/A	N/A	N/A
20	N/A	N/A	N/A	N/A
21	N/A	N/A	N/A	N/A
22	N/A	N/A	N/A	N/A
23	N/A	N/A	N/A	N/A
Average	186.5	6.9	142.5	0.049
SDEV	60.6	1.9	27.9	0.014
Min	129.0	4.8	98.0	0.030
Max	278.0	10.3	197.0	0.077

10 months				
**Animal #**	**dose (µCi)**	**dose (MBq)**	**SA (GBq/µmol)**	**IA (nmol)**
11	239	8.8	132	0.067
12	232	8.6	146	0.059
13	164	6.1	162	0.037
14	165	6.1	178	0.034
15	220	8.1	195	0.042
16	232	8.6	164	0.052
17	80	3.0	182	0.016
18	278	10.3	133	0.077
19	N/A	N/A	N/A	N/A
20	N/A	N/A	N/A	N/A
21	N/A	N/A	N/A	N/A
22	N/A	N/A	N/A	N/A
23	N/A	N/A	N/A	N/A
Average	201.3	7.4	161.5	0.048
SDEV	62.1	2.8	23.6	0.023

Min	80.0	3.0	133.0	0.016
Max	278.0	10.3	195.0	0.077

12 months				
**Animal #**	**dose (µCi)**	**dose (MBq)**	**SA (GBq/µmol)**	**IA (nmol)**
11	314	11.6	120	0.097
12	N/A	N/A	N/A	N/A
13	188	7.0	164	0.042
14	351	13.0	134	0.097
15	139	5.1	177	0.029
16	272	10.1	193	0.052
17	107	4.0	146	0.027
18	243	9.0	131	0.069
19	N/A	N/A	N/A	N/A
20	N/A	N/A	N/A	N/A
21	134	5.0	98	0.051
22	181	6.7	165	0.041
23	191	7.1	180	0.039
Average	212.0	7.8	150.8	0.054
SDEV	80.8	3.0	31.6	0.023
Min	107.0	4.0	98.0	0.027
Max	351.0	13.0	193.0	0.097

AD animals, blocking experiments

Animal #	dose (µCi)	dose (MBq)	SA (GBq/µmol)	IA (nmol)
24	163	6.0	184	0.033
25	147	5.4	196	0.028
24-blocking	151	5.6	NA	440
25-blocking	124	4.6	NA	440

WT animals, blocking experiments

Animal #	dose (µCi)	dose (MBq)	SA (GBq/µmol)	IA (nmol)
26	138	5.1	201	0.025
27	152	5.6	188	0.029
26-blocking	137	5.1	NA	440
27-blocking	115	4.3	NA	440

WT animals, PET biodistribution

3months				
**Animal**	**dose**	**dose**	**SA**	**IA**

#	(µCi)	(MBq)	(GBq/µmol)	(nmol)
28	155	5.7	184	0.031
29	123	4.6	175	0.026

Table S2. SUV values for [^{11}C]**4** uptake in different brain regions (average ± standard deviation).

	Cortex		STR	
	**WT**	**AD**	**WT**	**AD**
**4 months**	0.27 ± 0.04	0.31 ± 0.05	0.27 ± 0.05	0.32 ± 0.06
**6 months**	0.26 ± 0.07	0.35 ± 0.06	0.25 ± 0.07	0.34 ± 0.06
**8 months**	0.26 ± 0.09	0.36 ± 0.05	0.25 ± 0.08	0.36 ± 0.05
**10 months**	0.34 ± 0.06	0.30 ± 0.06	0.34 ± 0.07	0.28 ± 0.07
**12 months**	0.26 ± 0.05	0.24 ± 0.09	0.24 ± 0.06	0.24 ± 0.11

	Hippocampus		Cerebellum	
	**WT**	**AD**	**WT**	**AD**
**4 months**	0.27 ± 0.05	0.31 ± 0.05	0.27 ± 0.05	0.32 ± 0.06
**6 months**	0.25 ± 0.08	0.33 ± 0.06	0.24 ± 0.07	0.33 ± 0.07
**8 months**	0.24 ± 0.08	0.36 ± 0.06	0.23 ± 0.08	0.34 ± 0.06
**10 months**	0.32 ± 0.07	0.29 ± 0.07	0.32 ± 0.07	0.28 ± 0.06
**12 months**	0.25 ± 0.06	0.23 ± 0.10	0.25 ± 0.05	0.24 ± 0.11

	Brain Stem		Thalamus	
	**WT**	**AD**	**WT**	**AD**
**4 months**	0.28 ± 0.05	0.33 ± 0.06	0.27 ± 0.05	0.32 ± 0.05
**6 months**	0.26 ± 0.08	0.34 ± 0.07	0.24 ± 0.08	0.34 ± 0.07
**8 months**	0.25 ± 0.08	0.37 ± 0.05	0.23 ± 0.07	0.35 ± 0.06
**10 months**	0.32 ± 0.07	0.29 ± 0.07	0.32 ± 0.08	0.29 ± 0.07
**12 months**	0.25 ± 0.04	0.25 ± 0.10	0.25 ± 0.04	0.23 ± 0.11

Table S3. SUVr values for [^{18}F]florbetaben uptake in different brain regions (average ± standard deviation).

	Cortex		Hippocampus		Brain stem	
	**WT**	**AD**	**WT**	**AD**	**WT**	**AD**
**3 months**	0.86 ± 0.03	0.82 ± 0.06	1.03 ± 0.03	1.01 ± 0.05	1.17 ± 0.02	1.20 ± 0.04
**5 months**	0.89 ± 0.02	1.00 ± 0.06	1.01 ± 0.03	1.13 ± 0.04	1.18 ± 0.03	1.22 ± 0.03
**7 months**	0.84 ± 0.03	0.98 ± 0.07	1.01 ± 0.02	1.14 ± 0.04	1.18 ± 0.04	1.20 ± 0.03
**11 months**	0.88 ± 0.03	1.07 ± 0.07	0.98 ± 0.05	1.20 ± 0.06	1.22 ± 0.07	1.22 ± 0.05

1. Zhang W, Oya S, Kung MP, Hou C, Maier DL, Kung HF. F-18 Polyethyleneglycol stilbenes as PET imaging agents targeting Aβ aggregates in the brain. Nucl Med Biol. 2005; 32: 799-809.
2. Košak U, Brus B, Knez D, Šink R, Žakelj S, Trontelj J, et al. Development of an in-vivo active reversible butyrylcholinesterase inhibitor. Scientific Reports. 2016; 6.
3. Gómez-Vallejo V, Llop J. Fully automated and reproducible radiosynthesis of high specific activity [^{11}C]raclopride and [^{11}C]Pittsburgh compound-B using the combination of two commercial synthesizers. Nucl Med Commun. 2011; 32: 1011-7.
4. Ellman GL, Courtney KD, Andres Jr V, Featherstone RM. A new and rapid colorimetric determination of acetylcholinesterase activity. Biochem Pharmacol. 1961; 7: 88–90 ".

FINAL REPORT

eurofins DISCOVERY

In Vitro Pharmacology

Study of GUK-1402
Faculty of Pharmacy - University of Ljubljana Study Number: Eurofins02112020UK

STUDY ID: FR095-0022068

STUDY NUMBER
100055114_Euro-
fins02112020UK

November 27, 2020

CONFIDENTIAL

1. STUDY REFERENCES

**Study title**	***In Vitro* Pharmacology**<br>**Study of GUK-1402**<br>**Faculty of Pharmacy - University of LjubljanaStudy Number:Eurofins02112020UK**	
**Study number**	**100055114_Eurofins02112020UK**	**FINAL REPORT**<br>**November 27, 2020**
**Study ID**	**FR095-0022068**	
**Experimental period**	November 10, 2020 - November 26, 2020	
**Client personal study number**	Eurofins02112020UK	
**PO number**	3120/FK	

2. PERSONS INVOLVED IN THE STUDY

**Technical contact**	**Eurofins Cerep**<br>Le Bois l'Evêque<br>B.P. 30001<br>86 600 Celle l'Evescault<br>France	**Annie OTTO-BRUC,Ph.D.**<br>Principal Scientist, Pharmacology<br>AnnieOtto-Bruc@eurofins.com
**Study sponsor**	**Faculty of Pharmacy - University of Ljubljana**<br>Department of Medicinal Chemistry<br>Askerceva 7<br>1000 Ljubljana<br>SLOVENIA	**Damijan KNEZ**

3. APPROVAL

Head of laboratory statement

This study was conducted according to the procedures described in this report.

Eurofins Cerep
Le Bois l'Evêque
B.P. 30001
86 600 Celle l'Evescault
France

Sophie SEIGNEURIN,Mrs
Operations Director
SophieSeigneurin@eurofins.com

Signature

Quality assurance statement

This study was inspected by Eurofins Cerep Quality Control Unit, the results and methods presented in this report accurately reflect the methods used and the data collected for this study.

Eurofins Cerep
Le Bois l'Evêque
B.P. 30001
86 600 Celle l'Evescault
France

Eric BOUCHET
Quality Site Group Leader
EricBouchet@eurofins.com

Signature

4. TABLE OF CONTENTS

1. STUDY REFERENCES 2

2. PERSONS INVOLVED IN THE STUDY 2

3. APPROVAL 3

4. TABLE OF CONTENTS 4

5. SUMMARY 5

5.1. Study Design 5

5.2. Measurements 5

5.3. Results 5

6. COMPOUNDS 6

6.1. Test Compounds 6

6.2. Reference Compounds 6

7. RESULTS 7

7.1. *In Vitro* Pharmacology: Binding Assays 7

7.1.1. Test Compound Results 7

7.1.2. Reference Compound Results 11

7.2. *In Vitro* Pharmacology: Enzyme and Uptake Assays 14

7.2.1. Test Compound Results 14

7.2.2. Reference Compound Results 14

8. RESULTS INTERPRETATION GUIDE 15

9. MATERIALS AND METHODS 16

9.1. Experimental Conditions 16

9.1.1. *In Vitro* Pharmacology: Binding Assays 16

9.1.2. *In Vitro* Pharmacology: Enzyme and Uptake Assays 20

9.2. Analysis and expression of results 21

9.2.1. *In Vitro* Pharmacology: Binding Assays 21

9.2.2. *In Vitro* Pharmacology: Enzyme and Uptake Assays 21

10. BIBLIOGRAPHY 23

5. SUMMARY

The purpose of this study was to test GUK-1402 in Binding and enzyme and uptake assays.

5.1. Study Design

GUK-1402 was tested at 1.0E-05 M.

5.2. Measurements

Compound binding was calculated as a % inhibition of the binding of a radioactively labeled ligand specific for each target.

Compound enzyme inhibition effect was calculated as a % inhibition of control enzyme activity.

5.3. Results

Results showing an inhibition or stimulation higher than 50% are considered to represent significant effects of the test compounds.

Such effects were observed here and are listed in the following tables.

Assay	1.0E-05 M
α_{1A}*(h)* (antagonist radioligand)	71.7%
α_{2A}*(h)* (antagonist radioligand)	60.9%
D_{2S}*(h)* (agonist radioligand)	55.6%
M_1*(h)* (antagonist radioligand)	53.1%
M_2 *(h)* (antagonist radioligand)	85.7%
M_4*(h)* (antagonist radioligand)	63.2%
μ (MOP) *(h)* (agonist radioligand)	82.7%
5-HT_{1A}*(h)* (agonist radioligand)	96.2%
5-HT_{2A}*(h)* (agonist radioligand)	93%
5-HT_{2B}*(h)* (agonist radioligand)	88.4%
5-HT_6 *(h)* (agonist radioligand)	87.1%
5-HT_7 *(h)* (agonist radioligand)	84.3%
sigma 1 (h) (agonist radioligand)	99.9%
Potassium Channel hERG (human)- [3H] Dofetilide	84.8%
Na^+ channel (site 2) (antagonist radioligand)	99.1%
Lck kinase *(h)*	52.7%

6. COMPOUNDS

6.1. Test Compounds

Manufacturer: **Faculty of Pharmacy - University of Ljubljana**

Client Compound ID	Compound ID	Reference Number	Batch Number	FW	MW	Purity	Received Form	Stock solution	Flag
GUK-1402	100055114-1	-	#1	489.07	-	100.0	Powder	1.E-02 M DMSO	-

FW: Formula Weight - MW: Molecular Weight

6.2. Reference Compounds

In each experiment and if applicable, the respective reference compound was tested concurrently with GUK-1402, and the data were compared with historical values determined at Eurofins. The experiment was accepted in accordance with Eurofins validation Standard Operating Procedure.

7. RESULTS

7.1. *In Vitro* Pharmacology: Binding Assays

7.1.1. Test Compound Results

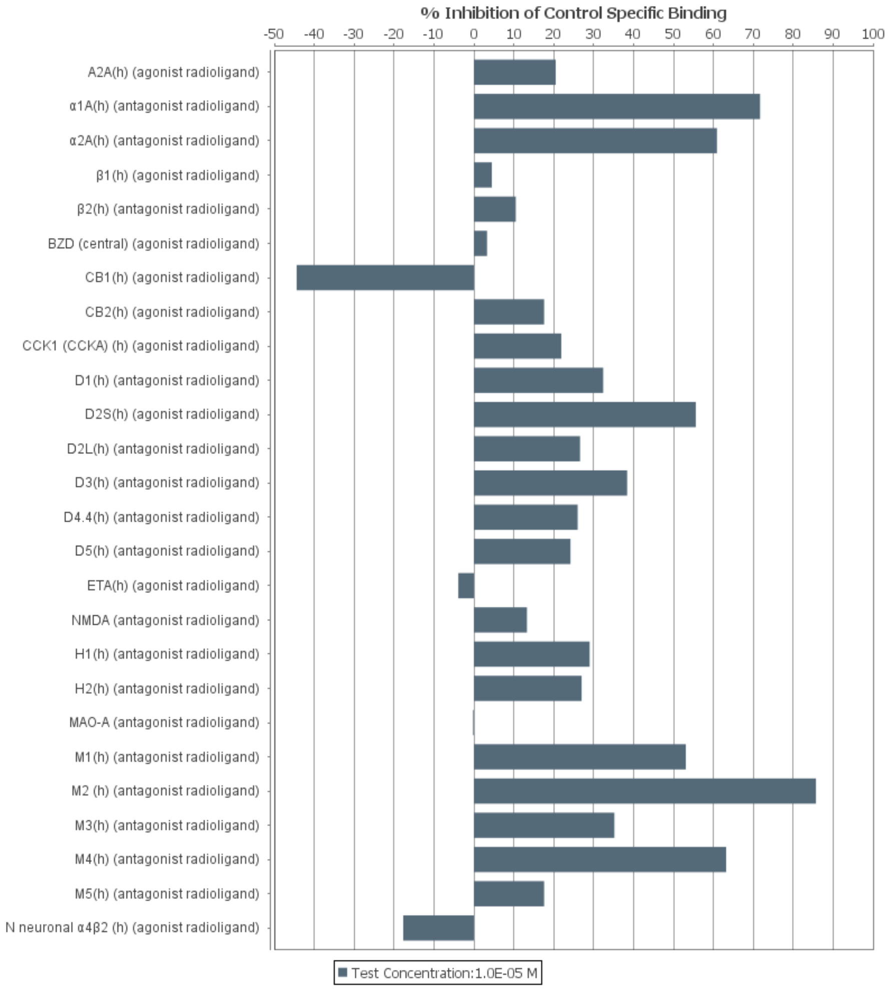

Figure 1. Histogram for GUK-1402 [1/2]

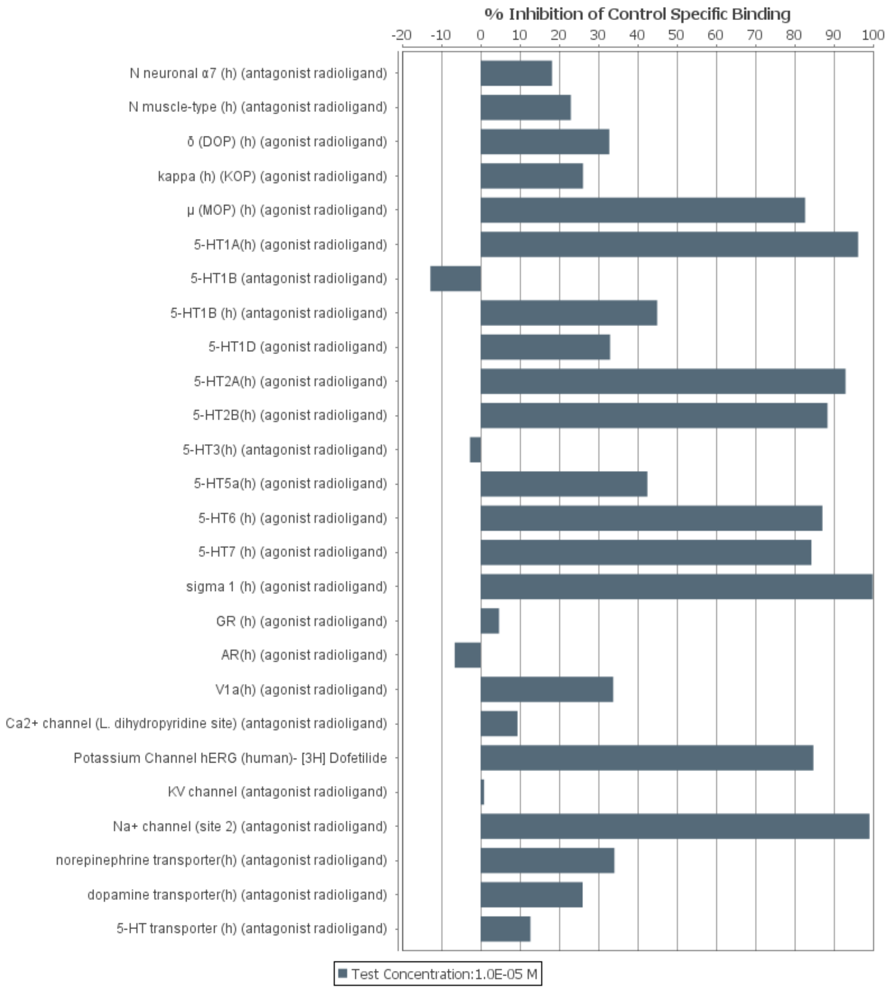

Figure 2. Histogram for GUK-1402 [2/2]

Compound I.D.	Client Compound I.D.	Test Concentration	% Inhibition of Control Specific Binding 1st	2nd	Mean
**A_{2A}*(h)* (agonist radioligand)**					
100055114-1	GUK-1402	1.0E-05 M	25.2	15.8	20.5
**α_{1A}*(h)* (antagonist radioligand)**					
100055114-1	GUK-1402	1.0E-05 M	72.2	71.1	**71.7**
**α_{2A}*(h)* (antagonist radioligand)**					
100055114-1	GUK-1402	1.0E-05 M	65.4	56.4	**60.9**
**β_1*(h)* (agonist radioligand)**					
100055114-1	GUK-1402	1.0E-05 M	-0.7	9.7	4.5
**β_2*(h)* (antagonist radioligand)**					
100055114-1	GUK-1402	1.0E-05 M	13.9	7.0	10.5
**BZD (central) (agonist radioligand)**					
100055114-1	GUK-1402	1.0E-05 M	1.9	4.8	3.3
**CB_1*(h)* (agonist radioligand)**					
100055114-1	GUK-1402	1.0E-05 M	-39.4	-49.4	-44.4
**CB_2*(h)* (agonist radioligand)**					
100055114-1	GUK-1402	1.0E-05 M	24.8	10.5	17.6

Compound I.D.	Client Compound I.D.	Test Concentration	% Inhibition of Control Specific Binding		
			1st	2nd	Mean
**CCK_1 (CCK_A) *(h)* (agonist radioligand)**					
100055114-1	GUK-1402	1.0E-05 M	27.2	16.6	21.9
**D_1*(h)* (antagonist radioligand)**					
100055114-1	GUK-1402	1.0E-05 M	39.0	25.7	32.4
**D_{2S}*(h)* (agonist radioligand)**					
100055114-1	GUK-1402	1.0E-05 M	55.7	55.5	**55.6**
**D_{2L}*(h)* (antagonist radioligand)**					
100055114-1	GUK-1402	1.0E-05 M	25.6	27.6	26.6
**D_3*(h)* (antagonist radioligand)**					
100055114-1	GUK-1402	1.0E-05 M	36.2	40.5	38.4
**$D_{4.4}$*(h)* (antagonist radioligand)**					
100055114-1	GUK-1402	1.0E-05 M	23.6	28.4	26.0
**D_5*(h)* (antagonist radioligand)**					
100055114-1	GUK-1402	1.0E-05 M	23.3	25.1	24.2
**ET_A*(h)* (agonist radioligand)**					
100055114-1	GUK-1402	1.0E-05 M	-3.0	-4.8	-3.9
**NMDA (antagonist radioligand)**					
100055114-1	GUK-1402	1.0E-05 M	8.4	18.3	13.3
**H_1*(h)* (antagonist radioligand)**					
100055114-1	GUK-1402	1.0E-05 M	28.3	29.7	29.0
**H_2*(h)* (antagonist radioligand)**					
100055114-1	GUK-1402	1.0E-05 M	27.7	26.4	27.0
**MAO-A (antagonist radioligand)**					
100055114-1	GUK-1402	1.0E-05 M	-0.5	0.1	-0.2
**M_1*(h)* (antagonist radioligand)**					
100055114-1	GUK-1402	1.0E-05 M	49.0	57.3	**53.1**
**M_2 *(h)* (antagonist radioligand)**					
100055114-1	GUK-1402	1.0E-05 M	85.5	85.8	**85.7**
**M_3*(h)* (antagonist radioligand)**					
100055114-1	GUK-1402	1.0E-05 M	33.8	36.6	35.2
**M_4*(h)* (antagonist radioligand)**					
100055114-1	GUK-1402	1.0E-05 M	63.6	62.8	**63.2**
**M_5*(h)* (antagonist radioligand)**					
100055114-1	GUK-1402	1.0E-05 M	18.6	16.7	17.6
**N neuronal α4β2 *(h)* (agonist radioligand)**					
100055114-1	GUK-1402	1.0E-05 M	-16.5	-18.9	-17.7
**N neuronal α7 *(h)* (antagonist radioligand)**					
100055114-1	GUK-1402	1.0E-05 M	16.1	20.3	18.2
**N muscle-type *(h)* (antagonist radioligand)**					
100055114-1	GUK-1402	1.0E-05 M	27.6	18.4	23.0
**δ (DOP) *(h)* (agonist radioligand)**					
100055114-1	GUK-1402	1.0E-05 M	31.5	34.1	32.8
**kappa *(h)* (KOP) (agonist radioligand)**					
100055114-1	GUK-1402	1.0E-05 M	30.0	22.3	26.1
**μ (MOP) *(h)* (agonist radioligand)**					
100055114-1	GUK-1402	1.0E-05 M	83.3	82.1	**82.7**
**5-HT_{1A}*(h)* (agonist radioligand)**					
100055114-1	GUK-1402	1.0E-05 M	96.8	95.6	**96.2**
**5-HT_{1B} (antagonist radioligand)**					
100055114-1	GUK-1402	1.0E-05 M	-17.0	-8.6	-12.8
**5-HT_{1B} *(h)* (antagonist radioligand)**					
100055114-1	GUK-1402	1.0E-05 M	50.7	39.3	45.0

Compound I.D.	Client Compound I.D.	Test Concentration	% Inhibition of Control Specific Binding 1st	2nd	Mean
**5-HT_{1D} (agonist radioligand)**					
100055114-1	GUK-1402	1.0E-05 M	29.8	36.2	33.0
**5-HT_{2A}*(h)* (agonist radioligand)**					
100055114-1	GUK-1402	1.0E-05 M	92.0	93.9	**93.0**
**5-HT_{2B}*(h)* (agonist radioligand)**					
100055114-1	GUK-1402	1.0E-05 M	88.5	88.3	**88.4**
**5-HT_{3}*(h)* (antagonist radioligand)**					
100055114-1	GUK-1402	1.0E-05 M	-3.4	-1.9	-2.7
**5-HT_{5a}*(h)* (agonist radioligand)**					
100055114-1	GUK-1402	1.0E-05 M	41.0	44.0	42.5
**5-HT_{6} *(h)* (agonist radioligand)**					
100055114-1	GUK-1402	1.0E-05 M	88.7	85.5	**87.1**
**5-HT_{7} *(h)* (agonist radioligand)**					
100055114-1	GUK-1402	1.0E-05 M	84.8	83.7	**84.3**
**sigma 1 (h) (agonist radioligand)**					
100055114-1	GUK-1402	1.0E-05 M	100.6	99.2	**99.9**
**GR *(h)* (agonist radioligand)**					
100055114-1	GUK-1402	1.0E-05 M	0.4	8.9	4.7
**AR(h) (agonist radioligand)**					
100055114-1	GUK-1402	1.0E-05 M	-2.8	-10.4	-6.6
**V_{1a}*(h)* (agonist radioligand)**					
100055114-1	GUK-1402	1.0E-05 M	33.7	33.8	33.8
**Ca^{2+} channel (L, dihydropyridine site) (antagonist radioligand)**					
100055114-1	GUK-1402	1.0E-05 M	9.5	9.2	9.4
**Potassium Channel hERG (human)- [3H] Dofetilide**					
100055114-1	GUK-1402	1.0E-05 M	87.3	82.3	**84.8**
**K_V channel (antagonist radioligand)**					
100055114-1	GUK-1402	1.0E-05 M	2.5	-0.8	0.9
**Na^+ channel (site 2) (antagonist radioligand)**					
100055114-1	GUK-1402	1.0E-05 M	99.8	98.5	**99.1**
**norepinephrine transporter*(h)* (antagonist radioligand)**					
100055114-1	GUK-1402	1.0E-05 M	26.8	41.5	34.1
**dopamine transporter*(h)* (antagonist radioligand)**					
100055114-1	GUK-1402	1.0E-05 M	22.9	29.1	26.0
**5-HT transporter *(h)* (antagonist radioligand)**					
100055114-1	GUK-1402	1.0E-05 M	13.5	11.8	12.7

7.1.2. Reference Compound Results

Compound I.D.	IC_{50}(M)	K_i(M)	nH
**A_{2A}*(h)* (agonist radioligand)**			
NECA	3.0E-08 M	2.5E-08 M	0.8
**α_{1A}*(h)* (antagonist radioligand)**			
WB 4101	6.1E-10 M	3.0E-10 M	1.8
**α_{2A}*(h)* (antagonist radioligand)**			
yohimbine	7.1E-09 M	3.1E-09 M	1.1
**β_1*(h)* (agonist radioligand)**			
atenolol	2.1E-07 M	1.2E-07 M	0.8
**β_2*(h)* (antagonist radioligand)**			
ICI 118551	1.3E-09 M	4.3E-10 M	0.9
**BZD (central) (agonist radioligand)**			
diazepam	1.6E-08 M	1.4E-08 M	0.9
**CB_1*(h)* (agonist radioligand)**			
CP 55940	4.8E-09 M	1.5E-09 M	1.0
**CB_2*(h)* (agonist radioligand)**			
WIN 55212-2	3.5E-09 M	2.3E-09 M	0.9
**CCK_1 (CCK_A) *(h)* (agonist radioligand)**			
CCK-8s	9.9E-11 M	7.4E-11 M	1.0
**D_1*(h)* (antagonist radioligand)**			
SCH 23390	5.0E-10 M	2.0E-10 M	1.1
**D_{2S}*(h)* (agonist radioligand)**			
7-OH-DPAT	2.9E-09 M	1.2E-09 M	0.9
**D_{2L}*(h)* (antagonist radioligand)**			
butaclamol	5.9E-09 M	1.5E-09 M	0.7
**D_3*(h)* (antagonist radioligand)**			
(+)butaclamol	5.5E-09 M	1.2E-09 M	1.1
**$D_{4.4}$*(h)* (antagonist radioligand)**			
clozapine	6.7E-08 M	2.6E-08 M	0.9
**D_5*(h)* (antagonist radioligand)**			
SCH 23390	5.3E-10 M	2.4E-10 M	0.9
**ET_A*(h)* (agonist radioligand)**			
endothelin-1	4.9E-11 M	2.4E-11 M	1.1
**NMDA (antagonist radioligand)**			
CGS 19755	2.5E-07 M	2.1E-07 M	0.8
**H_1*(h)* (antagonist radioligand)**			
pyrilamine	2.6E-09 M	1.7E-09 M	1.3
**H_2*(h)* (antagonist radioligand)**			
cimetidine	6.3E-07 M	6.1E-07 M	0.8
**MAO-A (antagonist radioligand)**			
clorgyline	1.8E-09 M	1.0E-09 M	1.5
**M_1*(h)* (antagonist radioligand)**			
pirenzepine	2.6E-08 M	2.3E-08 M	1.2
**M_2 *(h)* (antagonist radioligand)**			
methoctramine	5.9E-08 M	4.1E-08 M	0.9
**M_3*(h)* (antagonist radioligand)**			
4-DAMP	5.7E-10 M	4.1E-10 M	1.1
**M_4*(h)* (antagonist radioligand)**			
4-DAMP	9.8E-10 M	6.1E-10 M	1.3
**M_5*(h)* (antagonist radioligand)**			
4-DAMP	1.0E-09 M	5.0E-10 M	1.6

Compound I.D.	IC_{50}(M)	K_i(M)	nH
**N neuronal α4β2 *(h)* (agonist radioligand)**			
nicotine	9.3E-09 M	3.1E-09 M	0.8
**N neuronal α7 *(h)* (antagonist radioligand)**			
(±)epibatidine	8.6E-08 M	7.5E-08 M	1.2
**N muscle-type *(h)* (antagonist radioligand)**			
α-bungarotoxin	1.0E-09 M	8.3E-10 M	1.0
**δ (DOP) *(h)* (agonist radioligand)**			
DPDPE	2.8E-09 M	1.5E-09 M	1.0
**kappa *(h)* (KOP) (agonist radioligand)**			
U50488	6.9E-10 M	3.8E-10 M	1.1
**μ (MOP) *(h)* (agonist radioligand)**			
DAMGO	8.1E-10 M	3.3E-10 M	1.5
**$5\text{-}HT_{1A}$*(h)* (agonist radioligand)**			
8-OH-DPAT	1.9E-09 M	9.3E-10 M	>3
**$5\text{-}HT_{1B}$ (antagonist radioligand)**			
serotonin	2.8E-08 M	1.7E-08 M	0.8
**$5\text{-}HT_{1B}$ *(h)* (antagonist radioligand)**			
Serotonine	2.9E-07 M	1.3E-07 M	0.8
**$5\text{-}HT_{1D}$ (agonist radioligand)**			
serotonin	2.4E-09 M	8.1E-10 M	1.1
**$5\text{-}HT_{2A}$*(h)* (agonist radioligand)**			
(±)DOI	3.5E-10 M	2.6E-10 M	0.6
**$5\text{-}HT_{2B}$*(h)* (agonist radioligand)**			
(±)DOI	3.6E-09 M	1.8E-09 M	0.9
**$5\text{-}HT_3$*(h)* (antagonist radioligand)**			
MDL 72222	2.2E-08 M	1.5E-08 M	1.1
**$5\text{-}HT_{5a}$*(h)* (agonist radioligand)**			
serotonin	1.6E-07 M	7.8E-08 M	0.7
**$5\text{-}HT_6$ *(h)* (agonist radioligand)**			
serotonin	7.1E-08 M	3.3E-08 M	0.7
**$5\text{-}HT_7$ *(h)* (agonist radioligand)**			
serotonin	3.0E-10 M	1.1E-10 M	1.1
**sigma 1 (h) (agonist radioligand)**			
haloperidol	1.6E-09 M	8.5E-10 M	0.9
**GR *(h)* (agonist radioligand)**			
dexamethasone	4.5E-09 M	2.3E-09 M	1.5
**AR(h) (agonist radioligand)**			
testosterone	4.0E-09 M	1.8E-09 M	1.0
**V_{1a}*(h)* (agonist radioligand)**			
[d$(CH_2)_5^1$,Tyr$(Me)_2$]-AVP	1.5E-09 M	9.5E-10 M	1.1
**Ca^{2+} channel (L, dihydropyridine site) (antagonist radioligand)**			
nitrendipine	1.5E-10 M	9.8E-11 M	1.0
**Potassium Channel hERG (human)- [3H] Dofetilide**			
Terfenadine	5.6E-08 M	3.9E-08 M	0.9
**K_V channel (antagonist radioligand)**			
α-dendrotoxin	2.2E-10 M	1.8E-10 M	1.0
**Na^+ channel (site 2) (antagonist radioligand)**			
veratridine	6.1E-06 M	5.5E-06 M	0.9
**norepinephrine transporter*(h)* (antagonist radioligand)**			
protriptyline	4.3E-09 M	3.2E-09 M	1.1
**dopamine transporter*(h)* (antagonist radioligand)**			
BTCP	1.3E-08 M	6.7E-09 M	0.9

Compound I.D.	IC_{50}(M)	K_i(M)	nH
**5-HT transporter *(h)* (antagonist radioligand)**			
imipramine	2.7E-09 M	1.2E-09 M	1.0

7.2. *In Vitro* Pharmacology: Enzyme and Uptake Assays

7.2.1. Test Compound Results

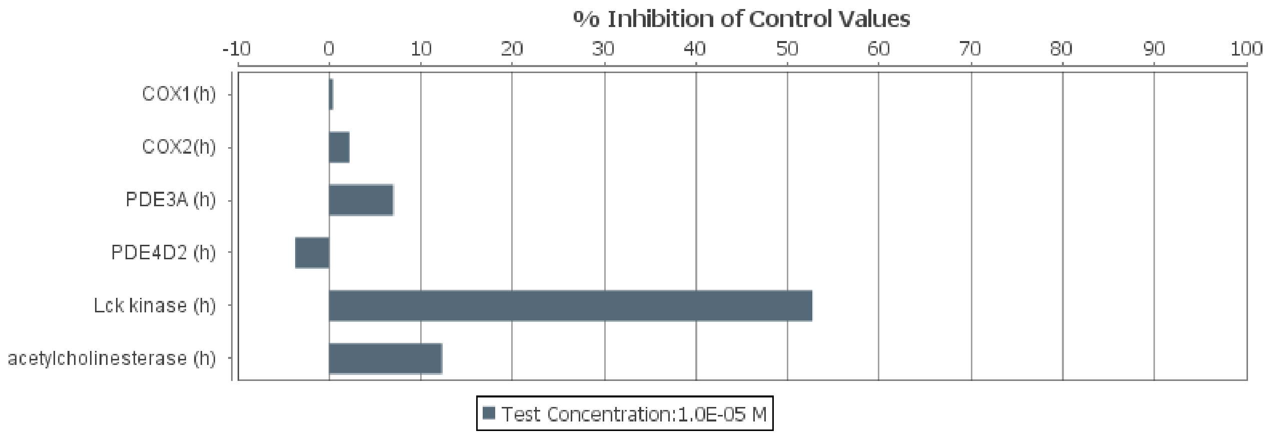

Figure 3. Histogram for GUK-1402

Compound I.D.	Client Compound I.D.	Test Concentration	% Inhibition of Control Values		
			1st	2nd	Mean
**COX1(h)**					
100055114-1	GUK-1402	1.0E-05 M	0.2	0.5	0.4
**COX2(h)**					
100055114-1	GUK-1402	1.0E-05 M	-1.8	6.2	2.2
**PDE3A *(h)***					
100055114-1	GUK-1402	1.0E-05 M	3.8	10.3	7.0
**PDE4D2 *(h)***					
100055114-1	GUK-1402	1.0E-05 M	-19.8	12.4	-3.7
**Lck kinase *(h)***					
100055114-1	GUK-1402	1.0E-05 M	58.4	47.1	**52.7**
**acetylcholinesterase *(h)***					
100055114-1	GUK-1402	1.0E-05 M	15.4	9.2	12.3

7.2.2. Reference Compound Results

Compound I.D.	IC_{50}(M)	nH
**COX1(h)**		
Diclofenac	1.7E-08 M	1.2
**COX2(h)**		
NS398	2.8E-07 M	1.5
**PDE3A *(h)***		
milrinone	5.8E-07 M	0.9
**PDE4D2 *(h)***		
Ro 20-1724	2.1E-07 M	0.7
**Lck kinase *(h)***		
staurosporine	2.5E-08 M	1.5
**acetylcholinesterase *(h)***		
galanthamine	5.9E-07 M	1.0

8. RESULTS INTERPRETATION GUIDE

In Vitro Pharmacology

Results showing an inhibition (or stimulation for assays run in basal conditions) higher than 50% are considered to represent significant effects of the test compounds. 50% is the most common cut-off value for further investigation (determination of IC_{50} or EC_{50} values from concentration-response curves) that we would recommend.
Results showing an inhibition (or stimulation) between 25% and 50% are indicative of weak to moderate effects (in most assays, they should be confirmed by further testing as they are within a range where more inter-experimental variability can occur).
Results showing an inhibition (or stimulation) lower than 25% are not considered significant and mostly attributable to variability of the signal around the control level.
Low to moderate negative values have no real meaning and are attributable to variability of the signal around the control level. High negative values (≥ 50%) that are sometimes obtained with high concentrations of test compounds are generally attributable to non-specific effects of the test compounds in the assays. On rare occasion they could suggest an allosteric effect of the test compound.

9. MATERIALS AND METHODS

9.1. Experimental Conditions

Minor variations to the experimental protocol described below may have occurred during the testing, they have no impact on the quality of the results obtained.

9.1.1. *In Vitro* Pharmacology: Binding Assays

Assay	Source	Ligand	Conc.	Kd	Non Specific	Incubation	Detection Method	Bibl.
**Receptors**								
**A_{2A} *(h)* (agonist radioligand)**	human recombinant (HEK-293 cells)	$[^3H]$CGS 21680	6 nM	27 nM	NECA (10 µM)	120 min RT	Scintillation counting	141
**α_{1A} *(h)* (antagonist radioligand)**	human recombinant (CHO cells)	$[^3H]$prazosin	0.1 nM	0.1 nM	epinephrine (0.1 mM)	60 min RT	Scintillation counting	897
**α_{2A} *(h)* (antagonist radioligand)**	human recombinant (CHO cells)	$[^3H]$RX 821002	1 nM	0.8 nM	(-)epinephrine (100 µM)	60 min RT	Scintillation counting	542
**β_1 *(h)* (agonist radioligand)**	human recombinant (HEK-293 cells)	$[^3H]$(-)CGP 12177	0.3 nM	0.39 nM	alprenolol (50 µM)	60 min RT	Scintillation counting	548
**β_2 *(h)* (antagonist radioligand)**	human recombinant (CHO cells)	$[^3H]$(-)CGP 12177	0.3 nM	0.15 nM	alprenolol (50 µM)	120 min RT	Scintillation counting	794
**CB_1 *(h)* (agonist radioligand)**	human recombinant (Chem-RBL cells)	$[^3H]$CP 55940	2 nM	0.9 nM	WIN 55212-2 (10 µM)	30 min 22°C	Scintillation counting	657
**CB_2 *(h)* (agonist radioligand)**	human recombinant (CHO cells)	$[^3H]$WIN 55212-2	0.8 nM	1.5 nM	WIN 55212-2 (5 µM)	120 min 37°C	Scintillation counting	165
**CCK_1 (CCK_A) *(h)* (agonist radioligand)**	human recombinant (CHO cells)	$[^{125}I]$CCK-8s	0.08 nM	0.24 nM	CCK-8s (1 µM)	60 min RT	Scintillation counting	562
**D_1 *(h)* (antagonist radioligand)**	human recombinant (CHO cells)	$[^3H]$SCH 23390	0.3 nM	0.2 nM	SCH 23390 (1 µM)	60 min RT	Scintillation counting	281
**D_{2S} *(h)* (agonist radioligand)**	human recombinant (HEK-293 cells)	$[^3H]$7-OH-DPAT	1 nM	0.68 nM	butaclamol (10 µM)	60 min RT	Scintillation counting	87
**D_{2L} *(h)* (antagonist radioligand)**	human recombinant (HEK-293 cells)	$[^3H]$methyl-spiperone	0.3 nM	0.1 nM	butaclamol (10 µM)	60 min RT	Scintillation counting	659
**D_3 *(h)* (antagonist radioligand)**	human recombinant (CHO cells)	$[^3H]$methyl-spiperone	0.3 nM	0.085 nM	(+)butaclamol (10 µM)	60 min RT	Scintillation counting	145
**$D_{4.4}$ *(h)* (antagonist radioligand)**	human recombinant (CHO cells)	$[^3H]$methyl-spiperone	0.3 nM	0.19 nM	(+)butaclamol (10 µM)	60 min RT	Scintillation counting	252
**D_5 *(h)* (antagonist radioligand)**	human recombinant (GH4 cells)	$[^3H]$SCH 23390	0.3 nM	0.25 nM	SCH 23390 (10 µM)	60 min RT	Scintillation counting	232
**ET_A *(h)* (agonist radioligand)**	human recombinant (CHO cells)	$[^{125}I]$endothelin-1	0.03 nM	0.03 nM	endothelin-1 (100 nM)	120 min 37°C	Scintillation counting	30

Assay	Source	Ligand	Conc.	Kd	Non Specific	Incubation	Detection Method	Bibl.
**H_1 *(h)* (antagonist radioligand)**	human recombinant (HEK-293 cells)	[^{3}H]pyrilamine	1 nM	1.7 nM	pyrilamine (1 µM)	60 min RT	Scintillation counting	492
**H_2 *(h)* (antagonist radioligand)**	human recombinant (CHO cells)	[^{125}I]APT	0.075 nM	2.9 nM	tiotidine (100 µM)	120 min RT	Scintillation counting	540
**M_1 *(h)* (antagonist radioligand)**	human recombinant (CHO cells)	[^{3}H]pirenzepine	2 nM	13 nM	atropine (1 µM)	60 min RT	Scintillation counting	59
**M_2 *(h)* (antagonist radioligand)**	human recombinant (CHO cells)	[^{3}H]AF-DX 384	2 nM	4.6 nM	atropine (1 µM)	60 min RT	Scintillation counting	59
**M_3 *(h)* (antagonist radioligand)**	human recombinant (CHO cells)	[^{3}H]4-DAMP	0.2 nM	0.5 nM	atropine (1 µM)	60 min RT	Scintillation counting	546
**M_4 *(h)* (antagonist radioligand)**	human recombinant (CHO cells)	[^{3}H]4-DAMP	0.2 nM	0.32 nM	atropine (1 µM)	60 min RT	Scintillation counting	59
**M_5 *(h)* (antagonist radioligand)**	human recombinant (CHO cells)	[^{3}H]4-DAMP	0.3 nM	0.3 nM	atropine (1 µM)	60 min RT	Scintillation counting	59
**N neuronal α4β2 *(h)* (agonist radioligand)**	human recombinant (SH-SY5Y cells)	[^{3}H]cytisine	0.6 nM	0.3 nM	nicotine (10 µM)	120 min 4°C	Scintillation counting	1084
**N neuronal α7 *(h)* (antagonist radioligand)**	human recombinant (SH-SY5Y cells)	[^{125}I]α-bungarotoxin	0.05 nM	0.34 nM	α-bungarotoxin (1 µM)	120 min 37°C	Scintillation counting	347
**N muscle-type *(h)* (antagonist radioligand)**	human endogenous (TE671 cells)	[^{125}I]α-bungarotoxin	0.5 nM	2 nM	α-bungarotoxin (5 µM)	120 min RT	Scintillation counting	524
**δ (DOP) *(h)* (agonist radioligand)**	human recombinant (Chem-1 (RBL) cells)	[^{3}H]DADLE	0.5 nM	0.6 nM	naltrexone (10 µM)	60 min RT	Scintillation counting	501
**kappa *(h)* (KOP) (agonist radioligand)**	human recombinant (RBL cells)	[3H]U69593	0.5 nM	0.6 nM	naloxone (10µM)	60 min RT	Scintillation counting	222
**µ (MOP) *(h)* (agonist radioligand)**	human recombinant (HEK-293 cells)	[^{3}H]DAMGO	0.5 nM	0.35 nM	naloxone (10 µM)	120 min RT	Scintillation counting	260
**5-HT_{1A} *(h)* (agonist radioligand)**	human recombinant (HEK-293 cells)	[^{3}H]8-OH-DPAT	0.5 nM	0.5 nM	8-OH-DPAT (10 µM)	60 min RT	Scintillation counting	164
**5-HT_{1B} (antagonist radioligand)**	rat cerebral cortex	[^{125}I]CYP (+ 30 µM isoproterenol)	0.1 nM	0.16 nM	serotonin (10 µM)	120 min 37°C	Scintillation counting	111
**5-HT_{1B} *(h)* (antagonist radioligand)**	human recombinant (Chem-1 (RBL) cells)	[3H]GR125743	1 nM	0.8 nM	Serotonine (10µM)	60 min 37°C	Scintillation counting	1451
**5-HT_{1D} (agonist radioligand)**	rat recombinant (CHO cells)	[^{3}H]serotonin	1 nM	0.5 nM	serotonin (10 µM)	60 min RT	Scintillation counting	777
**5-HT_{2A} *(h)* (agonist radioligand)**	human recombinant (HEK-293 cells)	[^{125}I](±)DOI	0.1 nM	0.3 nM	(±)DOI (1 µM)	60 min RT	Scintillation counting	288

Assay	Source	Ligand	Conc.	Kd	Non Specific	Incubation	Detection Method	Bibl.
**$5\text{-}HT_{2B}$ *(h)* (agonist radioligand)**	human recombinant (CHO cells)	$[^{125}I]$(±)DOI	0.2 nM	0.2 nM	(±)DOI (1 µM)	60 min RT	Scintillation counting	571
**$5\text{-}HT_{5a}$ *(h)* (agonist radioligand)**	human recombinant (HEK-293 cells)	$[^3H]$LSD	1.5 nM	1.5 nM	serotonin (100 µM)	120 min 37°C	Scintillation counting	193
**$5\text{-}HT_6$ *(h)* (agonist radioligand)**	human recombinant (CHO cells)	$[^3H]$LSD	2 nM	1.8 nM	serotonin (100 µM)	120 min 37°C	Scintillation counting	161
**$5\text{-}HT_7$ *(h)* (agonist radioligand)**	human recombinant (CHO cells)	$[^3H]$LSD	4 nM	2.3 nM	serotonin (10 µM)	120 min RT	Scintillation counting	217
**sigma 1 (h) (agonist radioligand)**	human endogenous (Jurkat cells)	$[^3H]$ (+)pentazocine	15 nM	16 nM	haloperidol (10 µM)	120 min 37°C	Scintillation counting	509
**GR *(h)* (agonist radioligand)**	human endogenous (IM-9 cells)	$[^3H]$dexametha sone	1.5 nM	1.5 nM	triamcinolone (10 µM)	6 hr 4°C	Scintillation counting	283
**AR(h) (agonist radioligand)**	human endogenous (LNCaP cells)	$[^3H]$methyltrien olone	1 nM	0.8 nM	testostérone (1 µM)	24 hr 4°C	Scintillation counting	498
**V_{1a} *(h)* (agonist radioligand)**	human recombinant (CHO cells)	$[^3H]$AVP	0.3 nM	0.5 nM	AVP (1 µM)	60 min RT	Scintillation counting	343
**Ion channels**								
**BZD (central) (agonist radioligand)**	rat cerebral cortex	$[^3H]$flunitrazepa m	0.4 nM	2.1 nM	diazepam (3 µM)	60 min 4°C	Scintillation counting	227
**NMDA (antagonist radioligand)**	rat cerebral cortex	$[^3H]$CGP 39653	5 nM	23 nM	L-glutamate (100 µM)	60 min 4°C	Scintillation counting	221
**$5\text{-}HT_3$ *(h)* (antagonist radioligand)**	human recombinant (CHO cells)	$[^3H]$BRL 43694	0.5 nM	1.15 nM	MDL 72222 (10 µM)	120 min RT	Scintillation counting	109
**Ca^{2+} channel (L, dihydropyridi ne site) (antagonist radioligand)**	rat cerebral cortex	$[^3H]$nitrendipine	0.1 nM	0.18 nM	nitrendipine (1 µM)	90 min RT	Scintillation counting	996
**Potassium Channel hERG (human)- [3H] Dofetilide**	human recombinant (HEK-293 cells)	[3H]Dofetilide	3 nM	6.6 nM	Terfenadine (25 µM)	60 min RT	Scintillation counting	1398
**K_V channel (antagonist radioligand)**	rat cerebral cortex	$[^{125}I]$α-dendrotoxin	0.01 nM	0.04 nM	α-dendrotoxin (50 nM)	60 min RT	Scintillation counting	225
**Na^+ channel (site 2) (antagonist radioligand)**	rat cerebral cortex	$[^3H]$batrachoto xin	10 nM	91 nM	veratridine (300 µM)	60 min 37°C	Scintillation counting	28
**Transporters**								
**norepinephrin e transporter *(h)* (antagonist radioligand)**	human recombinant (CHO cells)	$[^3H]$nisoxetine	1 nM	2.9 nM	desipramine (1 µM)	120 min 4°C	Scintillation counting	180
**dopamine transporter *(h)* (antagonist radioligand)**	human recombinant (CHO cells)	$[^3H]$BTCP	4 nM	4.5 nM	BTCP (10 µM)	120 min 4°C	Scintillation counting	190

Assay	Source	Ligand	Conc.	Kd	Non Specific	Incubation	Detection Method	Bibl.
**5-HT transporter *(h)* (antagonist radioligand)**	human recombinant (CHO cells)	$[^3H]$imipramine	2 nM	1.7 nM	imipramine (10 µM)	60 min RT	Scintillation counting	566
**Other enzymes**								
**MAO-A (antagonist radioligand)**	rat cerebral cortex	$[^3H]$Ro 41-1049	10 nM	14 nM	clorgyline (1 µM)	60 min 37°C	Scintillation counting	36

9.1.2. *In Vitro* Pharmacology: Enzyme and Uptake Assays

Assay	Source	Substrate/ Stimulus/Tracer	Incubation	Measured Component	Detection Method	Bibl.
**Kinases**						
**Lck kinase *(h)***	human recombinant (insect cells)	ATP + Ulight-Poly GAT[EAY(1:1:1)]n (25 nM)	10 min RT	phospho-Ulight-Poly GAT[EAY(1:1:1)]n	LANCE	556
**Other enzymes**						
**COX1(h)**	human recombinant	Arachidonic acid (3µM) + ADHP (25 µM)	3 min RT	Resorufin (oxydized ADHP)	Fluorimetry	1480
**COX2(h)**	human recombinant (Sf9 cells)	arachidonic acid (1.2 µM)+ ADHP (25 µM)	5 min RT	Resorufin (oxydized ADHP)	Fluorimetry	1480
**PDE3A *(h)***	human recombinant (Sf21 cells)	[3H]cAMP + cAMP (0.5µM)	15 min RT	[3H]5'AMP	Scintillation counting	1399
**PDE4D2 *(h)***	human recombinant (Sf9 cells)	[3H]cAMP + cAMP (0.5µM)	20 min RT	[3H]5'AMP	Scintillation counting	1399
**acetylcholinesteras e *(h)***	human recombinant (HEK-293 cells)	Acetylthiocholine (400 µM)	30 min RT	5 thio 2 nitrobenzoic acid	Photometry	63

9.2. Analysis and expression of results

9.2.1. *In Vitro* Pharmacology: Binding Assays

The results are expressed as a percent of control specific binding

$$\frac{\text{measured specific binding}}{\text{control specific binding}} * 100$$

and as a percent inhibition of control specific binding

$$100-\left(\frac{\text{measured specific binding}}{\text{control specific binding}} * 100\right)$$

obtained in the presence of GUK-1402.

The IC_{50} values (concentration causing a half-maximal inhibition of control specific binding) and Hill coefficients (nH) were determined by non-linear regression analysis of the competition curves generated with mean replicate values using Hill equation curve fitting

$$Y=D+\left[\frac{A-D}{1+(C/C_{50})^{nH}}\right]$$

where Y = specific binding, A = left asymptote of the curve, D = right asymptote of the curve, C = compound concentration, C_{50} = IC_{50}, and nH = slope factor. This analysis was performed using software developed at Cerep (Hill software) and validated by comparison with data generated by the commercial software SigmaPlot® 4.0 for Windows® (© 1997 by SPSS Inc.).
The inhibition constants (K_i) were calculated using the Cheng Prusoff equation

$$K_i=\frac{IC_{50}}{(1+L/K_D)}$$

where L = concentration of radioligand in the assay, and K_D = affinity of the radioligand for the receptor. A scatchard plot is used to determine the K_D.

9.2.2. *In Vitro* Pharmacology: Enzyme and Uptake Assays

The results are expressed as a percent of control specific activity

$$\frac{\text{measured specific activity}}{\text{control specific activity}} * 100$$

and as a percent inhibition of control specific activity

$$100-\left(\frac{\text{measured specific activity}}{\text{control specific activity}} * 100\right)$$

obtained in the presence of GUK-1402.

The IC_{50} values (concentration causing a half-maximal inhibition of control specific activity), EC_{50} values (concentration producing a half-maximal increase in control basal activity), and Hill coefficients (nH) were determined by non-linear regression analysis of the inhibition/concentration-response curves generated with mean replicate values using Hill equation curve fitting

$$Y=D+\left[\frac{A-D}{1+(C/C_{50})^{nH}}\right]$$

where Y = specific activity, A = left asymptote of the curve, D = right asymptote of the curve, C = compound concentration, C_{50} = IC_{50} or EC_{50} , and nH = slope factor.
This analysis was performed using software developed at Cerep (Hill software) and validated by comparison with data generated by the commercial software SigmaPlot® 4.0 for Windows® (© 1997 by SPSS Inc.).

10. BIBLIOGRAPHY

28. Brown, G.B. (1986), *J. Neurosci.,* 6*: 2064-2070.*
30. Buchan, K.W. et al. (1994), *Brit. J. Pharmacol.,* 112*: 1251-1257.*
36. Cesura, A.M. et al. (1990), *Mol. Pharmacol.,* 37*: 358-366.*
59. Dorje, F. et al. (1991), *J. Pharmacol. Exp. Ther.,* 256*: 727-733.*
63. Ellman, G.L. et al. (1961), *Biochem. Pharmacol.,* 7*: 88-95.*
87. Grandy, D.K. et al. (1989), *Proc. Natl. Acad. Sci. U.S.A.,* 86*: 9762-9766.*
109. Hope, A.G. et al. (1996), *Brit. J. Pharmacol.,* 118*: 1237-1245.*
111. Hoyer, D. et al. (1985), *Eur. J. Pharmacol.,* 118*: 1-12.*
141. Luthin, D.R. et al. (1995), *Mol. Pharmacol.,* 47*: 307-313.*
145. Mackenzie, R.G. et al. (1994), *Eur. J. Pharmacol.,* 266*: 79-85.*
161. Monsma, F.J. et al. (1993), *Mol. Pharmacol.,* 43*: 320-327.*
164. Mulheron, J.G. et al. (1994), *J. Biol. Chem.,* 269*: 12954-12962.*
165. Munro, S. et al. (1993), *Nature,* 365*: 61-65.*
180. Pacholczyk, T. et al. (1991), *Nature,* 350*: 350-354.*
190. Pristupa, Z.B. et al. (1994), *Mol. Pharmacol.,* 45*: 125-135.*
193. Rees, S. et al. (1994), *FEBS Lett.,* 355*: 242-246.*
217. Shen, Y. et al. (1993), *J. Biol. Chem.,* 268*: 18200-18204.*
221. Sills, M.A. et al. (1991), *Eur. J. Pharmacol.,* 192*: 19-24.*
222. Simonin, F. et al. (1995), *Proc. Natl. Acad. Sci. U.S.A.,* 92*: 7006-7010.*
225. Sorensen, R.G. and Blaustein, M.P. (1989), *Mol. Pharmacol.,* 36 *: 689-698.*
227. Speth, R.C. et al. (1979), *Life Sci.,* 24*: 351-358.*
232. Sunahara, R.K. et al. (1991), *Nature,* 350*: 614-619.*
252. Van Tol, H.H.M. et al. (1992), *Nature,* 358*: 149-152.*
260. Wang, J.B. et al. (1994), *FEBS Lett.,* 338*: 217-222.*
281. Zhou, Q.Y. et al. (1990), *Nature,* 347*: 76-80.*
283. Clark, A.F. et al. (1996), *Invest. Ophtalmol. Vis. Sci.,* 37*: 805-813.*
288. Bryant, H.U. et al. (1996), *Life Sci.,* 15*: 1259-1268.*
343. Tahara, A. et al. (1998), *Brit. J. Pharmacol.,* 125*: 1463-1470*.
347. Sharples, C.G.V. et al. (2000), *J. Neurosci.,* 20*: 2783-2791.*
492. Smit, M.J. et al. (1996), *Brit. J. Pharmacol.,* 117*: 1071-1080.*
498. Zava, D.T. et al. (1979), *Endocrinology,* 104*: 1007-1012.*
501. Simonin, F. et al. (1994), *Mol. Pharmacol.,* 46*: 1015-1021.*
509. Ganapathy, M.E. et al. (1999), *J. Pharmacol. Exp. Ther.,* 289*: 251-260.*
524. Lukas, R.J. (1986), *J. Neurochem.,* 46*: 1936-1941.*
540. Leurs, R. et al. (1994), *Brit. J. Pharmacol.,* 112*: 847-854.*
542. Langin, D. et al. (1989), *Eur. J. Pharmacol.,* 167*: 95-104.*
546. Peralta, E. G. et al. (1987), *Embo. J.,* 6*: 3923-3929.*
548. Levin, M.C. et al. (2002), *J. Biol.Chem.,* 277*: 30429-30435.*
556. Park, Y.M. et al. (1999), *Anal. Biochem.,* 269*: 94-104.*
562. Bignon, E. et al. (1999), *J. Pharmacol. Exp. Ther.* 289*: 742-751.*
566. Tatsumi, M. et al. (1999), *Eur. J. Pharmacol.,* 368*: 277-283.*
571. Choi, D.S. et al. (1994), *FEBS Lett.,* 352*: 393-399.*
657. Rinaldi-Carmona, M. et al. (1996), *J. Pharmacol. Exp. Ther.,* 278*: 871-878.*
659. Hall, D.A. and Strange, P.G. (1997), *Brit. J. Pharmacol.,* 121*: 731-736.*
777. Wurch, T. et al. (1997), *J. Neurochem.,* 68*: 410-418.*
794. Joseph, S.S. et al. (2004), *Naun.-Sch. Arch. Pharm.,* 369*: 525-532.*
897. Schwinn, D.A. et al. (1990), *J. Biol. Chem.,* 265*: 8183-8189.*
996. Gould, R.J. et al. (1982), *Proc. Natl. Acad. Sci. U S A.,* 79*: 3656-3660.*
1084. Gopalakrishnan, M. et al. (1996), *J. Pharmacol. Exp. Ther.,* 276*: 289-297.*
1398. Huang XP1, Mangano T, Hufeisen S, Setola V, Roth BL., *Assay Drug Dev Technol. 2010 Dec;8(6):727-42*
1399. Maurice D.H. et al. (2014), *Nat Rev Drug Discov.,* 13: *290-314*
1451. Maier DL, Sobotka-Briner C, Ding M, Powell ME, Jiang Q, Hill G, Heys JR, Elmore CS, Pierson ME and Mrzljak L (2009), Maier DL et al. J Pharmacol Exp Ther. 330(1):342, 2009.
1480. Pattaraporn Vanachayangkul and William H.Tolleson (2012), *Hindawi Publishing Corporation, Enzyme Research, Volume 2012, Article ID 416062, 7*
